# Retrotransposons spread potential *cis*-regulatory elements during mammary gland evolution

**DOI:** 10.1093/nar/gkz1003

**Published:** 2019-10-23

**Authors:** Hidenori Nishihara

**Affiliations:** Department of Life Science and Technology, Tokyo Institute of Technology, 4259-S2-17, Nagatsuta-cho, Midori-ku, Yokohama, Kanagawa 226-8501, Japan

## Abstract

Acquisition of *cis*-elements is a major driving force for rewiring a gene regulatory network. Several kinds of transposable elements (TEs), mostly retrotransposons that propagate via a copy-and-paste mechanism, are known to possess transcription factor binding motifs and have provided source sequences for enhancers/promoters. However, it remains largely unknown whether retrotransposons have spread the binding sites of master regulators of morphogenesis and accelerated *cis*-regulatory expansion involved in common mammalian morphological features during evolution. Here, I demonstrate that thousands of binding sites for estrogen receptor α (ERα) and three related pioneer factors (FoxA1, GATA3 and AP2γ) that are essential regulators of mammary gland development arose from a spreading of the binding motifs by retrotransposons. The TE-derived functional elements serve primarily as distal enhancers and are enriched around genes associated with mammary gland morphogenesis. The source TEs occurred via a two-phased expansion consisting of mainly L2/MIR in a eutherian ancestor and endogenous retrovirus 1 (ERV1) in simian primates and murines. Thus the build-up of potential sources for *cis*-elements by retrotransposons followed by their frequent utilization by the host (co-option/exaptation) may have a general accelerating effect on both establishing and diversifying a gene regulatory network, leading to morphological innovation.

## INTRODUCTION

Mammals share various common morphological features, but the evolutionary process responsible for the establishment of the *cis*-regulatory systems involved in their development is largely unknown. Transposable elements (TEs) make up a high proportion (30–50%) of mammalian genomes, most of which consist of retrotransposons (SINEs, LINEs and LTR-retrotransposons) that mobilize through RNA intermediates by a ‘copy-and-paste’ mechanism (1). TEs have in general been considered only as non-functional selfish DNA, parasitic elements or harmful mutagens (2–4). Because a vast majority of TEs has evolved neutrally (5), they are considered to have no advantageous effect on the host; however, a part of functional sequences such as protein-coding exons and gene regulatory elements were derived from TEs (co-option or exaptation) (6). Notably, several TE copies under purifying selection have acquired enhancer/promoter functions for developmental genes involved in morphological evolution in mammals (7–11). These examples, however, explain only a small fraction of the TE population, and it remains unknown how large numbers of TEs have been co-opted and have contributed to the evolution of morphological novelties in mammals.

Several kinds of retrotransposons are known to possess transcription factor binding motifs, and many copies of these serve most often as enhancers (12–19), suggesting that retrotransposons can amplify potential source sequences for *cis*-elements (20). This raises the further possibility that if retrotransposons amplified potential binding motifs of master developmental regulators—rather than the case involving cut-and-paste DNA transposons (21)—a large number of TE-derived enhancers/promoters might facilitate a dramatic increase in downstream genes of the regulators and the organization of a gene regulatory network involved in morphological evolution. Little is known, however, about whether retrotransposons contributed in such a way to the acquisition of common morphological features in mammals such as the mammary gland.

**Figure 1.**
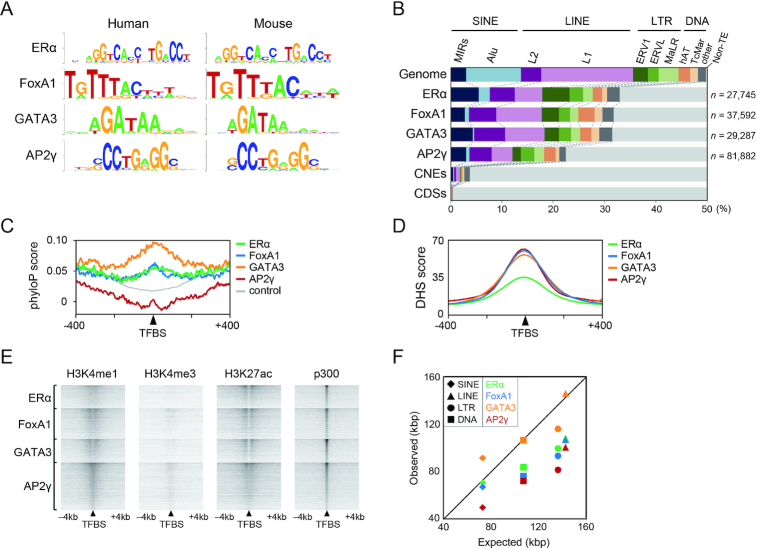
Profile of TEs associated with ERα, FoxA1, GATA3 and AP2γ. (**A**) Binding motifs of the four transcription factors in human and mouse genomes retrieved from HOCOMOCO (75). (**B**) Percentage of TEs among the number of binding events (*n*) for ERα, FoxA1, GATA3 and AP2γ. Proportions of TEs in the human genome (hg19, excluding chromosome Y) (Genome), conserved non-coding elements (CNEs), and protein-coding sequences (CDSs) are shown. SINE, short interspersed element; LINE, long interspersed element; LTR, long terminal repeat retrotransposon; DNA, DNA transposons. (**C**) Average phyloP conservation scores around the TE-associated binding sites (±400 bp). TFBS, transcription factor binding site. (**D**) DNase I hypersensitive sequence (DHS) score around the TE-associated binding sites (±400 bp). (**E**) ChIP-seq heat maps for histone modification and p300 signals around the TE-associated TFBSs (±4 kb; normalized to 8M reads). (**F**) Average distances from the TE-derived TFBSs to the nearest transcription start site (TSS), as compared with those from random sites in all TEs in the human genome (expected; see Materials and Methods).

The mammary gland is, as the name suggests, an organ that differentiates Mammalia from other animals. The mammary gland is considered to have evolved from an ancestral apocrine-like gland, and secretions from mammary patches might have occurred during the early evolution of synapsids (extinct amniotes closely related to mammals) (22). Molecular and developmental studies have revealed that cell fate determination and initial development of the mammary gland require binding of ERα to many enhancers and promoters to activate the expression of related genes (23–25). A few transcription factors, called pioneer factors, such as FoxA1, GATA3 and AP2γ, have pivotal roles in addition to ERα in a dramatic change in gene expression during early mammary gland development (25–27). These genes also exist in non-mammalian vertebrates. Their binding sites are unchanged across humans and mice (Figure 1A). Therefore, acquisition of *cis*-elements bound by these transcription factors is expected to have contributed to alteration of gene regulatory networks involved in mammary gland evolution, and a subset of which might result from a spreading of their potential binding sites via (retro-)transposition of TEs. Here, I explored co-opted mammalian TEs bound by the four transcription factors and demonstrate that thousands of TEs have accelerated a two-phased *cis*-regulatory expansion by spreading these potential binding sites during mammalian evolution.

## MATERIALS AND METHODS

### ChIP-seq data analysis

ChIP-seq data from ERα-positive human mammary MCF-7 cells were used in this study. Although MCF-7 is used as a model cell line for breast cancer, basic binding sites for ERα, FoxA1, GATA3 and AP2γ during cell proliferation can be assumed to be conserved to some extent from the normal mammary gland cells because they are expressed in both the normal and MCF-7 cells. Raw ChIP-seq data from estrogen-treated MCF-7 cells for ERα, FoxA1, GATA3 and AP2γ antibodies were obtained from the NCBI SRA database (28–30; Supplementary Table S2). Bowtie version 1.1.2 (31) was used to map the ChIP-seq reads on the human hg19 genome. Reads with multiple hits were discarded to exclude false detection of non-specific repetitive sequences (-m 1 option). Peak calling was carried out with MACS2 ver. 2.1.0 (32,33) with a false discovery rate *q*-value of <0.01, in which summits of subpeaks were also provided.

### Detection of TE-associated ChIP-seq peaks

RepeatMasker (version open-4.0.6) (http://www.repeatmasker.org/) was used for TE annotation of the human hg19 genome with the cross_match engine and the sensitive (-s) option. The consensus sequences of MER49_4D and LTR8C, which are newly characterized ERV1 elements described in this study, were added to the default human repeat library (version 20160829). Satellite DNAs, simple repeats, and small RNAs were excluded from consideration in this study. Age distributions of the TEs were estimated based on the sequence divergence of each TE copy from the consensus as retrieved from RepeatMasker.

By comparing the genomic position of the ChIP-seq (sub-)peak summits and the repeat annotation, the number of TE-associated binding sites for the four transcription factors was determined. The proportion of each TE family in the human genome was calculated excluding chromosome Y data to take into account the female-derived MCF-7 line. Based on the fraction of TEs, the number of TE-associated binding sites was evaluated with a two-tailed binomial test in R, followed by a correction of the original *P*-values with the Bonferroni method (*n* = 2144).

### Analysis for the transcription factor binding sites within TEs

For each of the ChIP-seq peak summits located within a TE, its genomic position was converted to a site position corresponding to the consensus sequence of the TE subfamily by referring to the repeat alignment (.align output of RepeatMasker). The distribution of the binding sites was visualized with a dot-plot by converting the length of the TE consensus sequence to 100 bins. The number of binding events on TEs was normalized based on the total number of TE copies for each site of the subfamilies calculated from the RepeatMasker output. The 100 bins were separated into ten equal-length segments containing ten bins each, and a uniform distribution of the binding sites among the segments was tested with Fisher's exact test or χ^2^-test if the number of binding events was 30–100 or >100 for a TE subfamily, respectively. The original *P*-values were corrected with the Bonferroni method (*n* = 278). If the binding sites showed a significant non-uniform distribution within the TE consensus sequence (*P* < 0.05), the FIMO (34) tool was used to test whether the binding motifs of the four transcription factors obtained from the JASPAR database (35) are present in each binding peak region of the consensus sequence. The TE sequences having the binding sites in each peak were extracted and aligned with MAFFT (36) with the most accurate setting (-localpair, -maxiterate 1000), and the sequence motifs were illustrated by WebLogo (37).

### Analysis of evolutionary conservation and DNase I hypersensitive sites (DHSs) for the TE-associated binding regions

The per-site conservation scores (hg19.100way.phyloP100way) (38) were obtained from the UCSC Genome Browser database (39). Average conservation scores per site were calculated for the ±400 bp flanking regions of the ChIP-seq peak summits for ERα, FoxA1, GATA3 and AP2γ in the TEs, and 10-bp moving average was visualized. As a control, 1 000 000 random sites were chosen from the human genome, and 457 960 sites overlapped with TEs were used for the same calculation. For DHS analysis, ENCODE data generated by the University of Washington were obtained from the UCSC Genome Browser database (http://hgdownload.cse.ucsc.edu/goldenPath/hg19/encodeDCC/wgEncodeUwDnase/), which provides DHSs with a 20-bp window for MCF-7 cells that were treated with 100 nM 17β-estradiol. Average DHS scores per the 20-bp window were calculated for the ±400 bp flanking regions of the binding sites in TEs for the four transcription factors.

### Proportion of TEs in protein-coding sequences (CDSs) and conserved non-coding elements (CNEs)

Annotation data for CDS in human (hg19) and mouse (mm10) genomes were retrieved from the refFlat files in the UCSC Genome Browser database. Based on the RepeatMasker output, proportions of each family of TEs were calculated with the exclusion of Y chromosome data. Conserved elements that evolved under purifying selection were identified based on a length of >20 bp and a lod score of >60 as retrieved from the UCSC phastCons elements data for human and mouse (phastConsElements100way and phastConsElements60way, respectively). CNE lists in human and mouse were obtained by removing the CDS regions identified above from the conserved element regions. The proportion of each family of TEs in the CNEs was calculated in the same way as above.

### Distances between TEs and transcription start sites (TSSs)

Average distances between the TE-associated binding sites and the nearest TSS based on the UCSC Gene annotation were calculated separately for the four TE classes (SINEs, LINEs, LTR-retrotransposons and DNA transposons) for each of the four transcription factors (ERα, FoxA1, GATA3 and AP2γ). As a control, 1,000,000 random sites were chosen from the human genome, and average distances between the nearest TSS and 126 401, 206 610 88 094 and 35 010 sites overlapping with the SINEs, LINEs, LTR-retrotransposons and DNA transposons, respectively, were compared.

### Chromatin states of the TE-associated binding sites

Histone H3 lysine 4 monomethylation (H3K4me1), histone H3 lysine 4 trimethylation (H3K4me3), and histone H3 lysine 27 acetylation (H3K27ac) are hallmark histone modifications for enhancers, promoters, and active chromatin states, respectively (40). The MCF-7 histone marks of H3K4me1, H3K4me3 and H3K27ac, as well as the p300 binding states, were obtained from the NCBI SRA database (Supplementary Table S2) and used to estimate the functions of the TE-associated binding sites of the four transcription factors. Mapping and peak calling were conducted as described above. From each set of antibody data, 8 000 000 uniquely mapped ChIP-seq reads were randomly selected for normalization. The chromatin states around the binding sites of the four transcription factors (±4 kb) were visualized as heat maps using EaSeq (41).

### Colocalization of multiple factors within the same TEs

Colocalization of two transcription factors was evaluated for any combination among ERα, FoxA1, GATA3, AP2γ and p300, in which it was tested whether a certain TE sequence bearing one kind of transcription factor binding site tended to be bound by another factor. The TEs bound by the factors were categorized among superfamilies/clades, and those showing >10 binding events per the category were used for the analysis. Among all the 4 525 412 TE copies in the human genome, 9005 (0.17%), 11 730 (0.22%), 9027 (0.17%), 18 045 (0.35%) and 4530 (0.087%) were on average bound by ERα, FoxA1, GATA3, AP2γ and p300, respectively. The average binding probabilities per TE superfamilies/clades were used as a control. For every combination of two from the five factors, enrichment of TEs bound by one factor under the condition of the other factor binding was evaluated by the χ^2^-test.

### Functional annotation of the transcription factor binding elements

Annotation for functions of the MCF-7 genomic segments was performed with ChromHMM (42). Model learning was conducted with H3K4me1, H3K4me3 and H3K27ac histone marks, as well as association of the CTCF insulator protein in MCF-7 cells (Supplementary Table S2). The genomic regions were annotated with eight states according to the distribution of the histone marks (with H3K4me1, H3K4me3 and H3K27ac representing marks for enhancers, promoters, and active/positive regulatory regions, respectively) and CTCF binding (indicating insulators) as well as the enrichment in various genomic annotations, such as the distance from TSSs and overlap with CpG islands (see Supplementary Figure S2). After the estimation, the TE and non-TE binding sites for ERα, FoxA1, GATA3 and AP2γ were separately evaluated.

### Luciferase reporter assay

A human L2 sequence (chr12:102 942 384–102 942 727; hg19) bound by ERα was amplified by PCR by using the Phusion Hot Start II DNA Polymerase kit (Thermo Fisher Scientific) and specific primers (Forward: 5′-AAGAAAGAAAAAAAGCAAAT-3′, and Reverse: 5′-AGTTGGAAAGAAGGATAGAT-3′). The orthologous sequences from the common marmoset and African elephant were synthesized by gBlocks Gene Fragments (Integrated DNA Technologies) by referring to the genome sequences of chr9:92 628 881–92 629 221 (calJac3) and scaffold_2:98 064 003–98 064 340 (loxAfr3) in the UCSC Genome Browser, respectively. The DNA fragments were inserted into the pNL3.1 reporter vector containing a minimal promoter and *Oplophorus* luciferase (Promega), followed by confirmation by sequencing. For the mutation construct, the ERα-binding motif ‘AGGTCANNNTGACCT’ in the human sequence was changed to ‘TCCAGTNNNACTGGA’ and used for the assay. The MCF-7 cells, which tested mycoplasma negative, were purchased from the Japanese Collection of Research Bioresources Cell Bank and maintained with Dulbecco's modified Eagle's medium (DMEM) (Thermo Fisher Scientific) supplemented with 10% fetal bovine serum (FBS; Nichirei). Cells were plated in 24-well plates with phenol red–free DMEM (Thermo Fisher Scientific) supplemented with 5% dextran-coated charcoal-treated FBS (Thermo Fisher Scientific) and 4 mM l-glutamine (Wako) for 48 h. Transient co-transfection was conducted using FuGene HD (Promega) for 48 h with 100 ng of the test plasmid as well as 50 ng of pGL4.53 firefly luciferase vector for an internal control. A human non-functional genomic sequence (chr3:30 000–30 200; hg38) was used as a negative control. The cells were treated with 100 nM 17β-estradiol (Sigma) for 3 h prior to assays. Luciferase reporter assays were conducted using Nano-Glo Dual-Luciferase Reporter Assay kit (Promega) with the cell lysate prepared by Passive Lysis Buffer (Promega) according to the manufacturer's instructions. All experiments were performed with three biological replicates and evaluated with a two-tailed *t*-test.

### Ortholog search for the binding sequences among amniotes

Orthologous sequences for the binding sites of ERα, FoxA1, GATA3 and AP2γ were searched with the liftOver tool using the cross-species chain data obtained from the UCSC Genome Browser database (39). This tool was selected because the chain data contains information for long (megabase-scale) alignments of orthologous regions even for TE sequences that was integrated over one million years ago (Mya). However, there was the possibility of missing their orthologs because of species-specific missing (unassembled) data or large deletions in TEs. To minimize this possibility, This search was carried out against multiple species in each clade if possible. The 10-bp regions around all the ChIP-seq summits of the human genome (hg19) were used as input. Orthologs were searched in the following amniote species: chimpanzee (PanTro4), gorilla (GorGor3), orangutan (PonAbe2), gibbon (NomLeu3), baboon (PapAnu2 and PapHam1), rhesus monkey (RheMac3), marmoset (CalJac3), tarsier (TarSyr1), mouse lemur (MicMur1), bush baby (OtoGar1), tree shrew (TupBel1), Chinese hamster (CriGri1), guinea pig (CavPor3), squirrel (SpeTri1), kangaroo rat (DipOrd1), mouse (Mm10), rat (Rn6), rabbit (OryCun2), pika (OchPri3), horse (EquCab2), white rhinoceros (CerSim1), microbat (MyoLuc1), megabat (PteVam1), dog (CanFam3), panda (AilMel1), cat (FelCat5), dolphin (TurTru1), cow (BosTau7), sheep (OviAri3), pig (SusScr2), alpaca (VicPac1), hedgehog (EriEur2), shrew (SorAra2), sloth (ChoHof1), armadillo (DasNov2), elephant (LoxAfr3), rock hyrax (ProCap1), tenrec (EchTel2), wallaby (MacEug1), Tasmanian devil (SarHar1), opossum (MonDom5), platypus (OrnAna1), chicken (GalGal3), turkey (MelGal1), zebra finch (TaeGut2), American alligator (AllMis1) and Anolis lizard (AnoCar2). For the analysis of the orthologous sequences of TE-associated binding sites, the known limited distribution of each TE subfamily retrieved from the RepeatMasker library was taken into account. Because the human loci were used as queries for searches in other animals, a classification of human TEs was applicable to all TEs inserted in the lineages leading to humans. Among the species where orthologs were detected, the most distantly related species from humans on the reference phylogenetic tree (43–48) was determined for each binding event.

### ChIP-seq data analysis for ERα-binding sites in the mouse mammary gland

The ChIP-seq data for ERα from the mouse mammary gland (49; NCBI GEO: GSE43415) were reanalyzed. ChIP-seq peaks were called in the same way described above with the mm10 mouse genome. Satellite DNAs, simple repeats, and small RNAs were excluded from consideration. Orthologous sequences for the ERα binding regions were searched with the liftOver tool using the following cross-species chain data: rat (Rn6), kangaroo rat (DipOrd2), guinea pig (CavPor3), naked mole rat (HetGla2), squirrel (SpeTri2), pika (OchPri2), rabbit (OryCun2), human (Hg19), cow (BosTau8), dog (CanFam3), rhinoceros (CerSim1), horse (EquCab2), sloth (ChoHof1), armadillo DasNov3), tenrec (EchTel2), elephant (LoxAfr3), hyrax (ProCap1), wallaby (MacEug2), Tasmanian devil (SarHar1), opossum (MonDom5), platypus (OrnAna1), chicken (GalGal5) and Anolis lizard (AnoCar2). The subsequent analysis was performed in the same way as described above. Among the species where orthologs were detected, the most distantly related species from mouse on the reference phylogenetic tree (48,50,51) was determined for each binding events.

### Gene ontology analysis for neighboring genes of the TEs

To estimate the biological significance of the TE-derived functional elements, gene ontology analyses for their neighboring genes were conducted with GREAT version 3.0 (52) with the basal plus extension option. This analysis was conducted for TEs annotated as active promoters or strong enhancers with ChromHMM and bound by ERα (*n* = 1453), FoxA1 (*n* = 1726), GATA3 (*n* = 1186), AP2γ (*n* = 2882) and p300 (*n* = 1461). A false discovery rate *q*-value of <0.05 and >2-fold enrichment (observed relative to expected) was used for visualization.

### Genome-wide density of TEs bound by the transcription factors

The genomic distribution of TE density (copies per kilobase) bound by any of the four factors, ERα, FoxA1, GATA3 and AP2γ, was calculated with a sliding 100-kb window with 50-kb steps across the entire human genome (hg19 except chromosome Y). For chromosome 20, this distribution was calculated with a sliding 100-kb window with 10-kb steps. The lists of protein-coding RefSeq genes and TE classes were retrieved from the UCSC Table browser.

## RESULTS

### Thousands of TEs bound by ERα, FoxA1, GATA3 and AP2γ mostly show the signature of enhancers

To identify TE-derived binding sites of transcription factors involved in mammary gland evolution, I thoroughly explored the known genome-wide binding sites for ERα and the three pioneer factors in estrogen-positive human mammary epithelial cells (MCF-7) and their association with all kinds of TEs. For comparison, the proportion of TEs in protein coding sequences (CDSs) and conserved non-coding elements (CNEs) that are under purifying selection was found to be only 0.4% and 3.7%, respectively (Figure 1B), suggesting a general view that most TEs rarely contribute to putative functional sequences in spite of their large fraction in the genome (48%). Contrary to this, among the ChIP-seq peaks, 32.9%, 31.7%, 31.4% and 22.5% were derived from TEs for ERα, FoxA1, GATA3 and AP2γ, respectively (Figure 1B). In total 38 500 TEs harbor at least one of the binding sites, and the TEs bound by each of the four factors were classified into TE categories. Whereas a large fraction of the human genome consists of *Alu* (10.7%) and L1 (17.9%) sequences, the percentages of these sequences among the binding sites were very low (e.g. 2.2% and 5.4% for ERα, respectively; Figure 1B). Although at least two-thirds of the binding sites were derived from non-TE sequences, it should be noted that larger percentages of them are represented by MIR SINE, L2 LINE, and endogenous retrovirus (ERV1 and ERVL) sequences relative to CDSs/CNEs and even to the human genome. For example, MIR, L2, ERV1 and ERVL represents 5.4%, 4.9%, 5.3% and 2.6% of the ERα-binding sites, whereas they occupy 3.0%, 3.9%, 2.9% and 2.0% of the human genome (Figure 1B), respectively. Although these increased percentages among the binding sites of all transcription factors were not seen for all types of TEs, this result may suggest a greater contribution by specific retrotransposon types to the acquisition of the binding sites during evolution, which will be addressed later.

Evolutionary conservation of the binding sites in TEs was evaluated (Figure 1C). The PhyloP conservation scores in TEs are in general lower than those in non-TE sequences because of their nature of presence in limited species, and therefore the scores tend to be increased in the flanking sequence of TEs (as shown by the control in Figure 1C). Nevertheless, the average scores for the TE-derived binding sites of ERα, FoxA1, and GATA3 were 0.064, 0.067 and 0.098, respectively, which were significantly greater than random TEs (0.017 in control; *P* < 10^−7^, two-tailed *t*-test), while the AP2γ-binding sites showed no significant difference. Although the increases of the average conservation scores are not very high, this result suggests that a part of the TEs bound by the three factors are evolutionarily conserved (*i.e*., under purifying selection), as shown by representative loci in Supplementary Figures S17–S20. In addition, the DHS scores that represent open chromatin accessibility are higher around the binding sites (Figure 1D). The binding sites on TEs exhibited high levels of H3K4me1 and H3K27ac marks as well as a very low signal for H3K4me3, indicating the typical chromatin signature of enhancers (Figure 1E). Indeed, all four factors as well as the transcriptional co-activator p300 tended to colocalize within the same TE sequence (113- to 254-fold enrichment; Supplementary Figure S1). The TEs associated with ERα, FoxA1 and AP2γ showed enrichment in genomic locations closer to TSSs (Figure 1F). These findings suggest that the TE-associated binding regions serve primarily as distal enhancers rather than promoters. This conclusion is also supported by a chromatin state classification by ChromHMM (42), whereby 31.1% and only 1.6% of the TE-associated binding sites were classified as enhancers and promoters, respectively, whereas enhancers and promoters account for 38.7% and 9.8%, respectively, of the non-TE-associated binding sites (Supplementary Figure S2). In addition, 62.0% of the TEs bound by all four factors were classified as enhancers, whereas promoters account for only 1.1% of them (Supplementary Figure S2E), suggesting their major contribution as enhancers. Therefore, although some TEs are known to have been co-opted as promoters (53), a very small proportion of co-opted TEs act as promoters in the cases of these four transcription factors.

Next, I determined whether the binding sites are localized on certain kinds of TEs for all 536 TE families in humans. A significant enrichment of the transcription factor associations within a total of 55 TE families (5 SINEs, 5 LINEs, 32 LTR-retrotransposons and 13 DNA transposons), including known cases of ERα binding to MIRs and MER41 (12,54), was found (Supplementary Figure S3). For example, the binding sites for ERα, FoxA1, GATA3 and AP2γ were all significantly enriched in L2 LINEs (*P* < 10^−12^, *P* < 10^−34^, *P* < 10^−66^ and *P* < 10^−5^, respectively; two-tailed binomial test with the Bonferroni correction). Although most TE families (481 of the 536 families) showed no significant enrichment for the binding sites of any transcription factor (Supplementary Figure S3), it should be noted that some members of the other 55 TE families might have spread their potential binding sites via (retro-)transposition.

### Dozens of retrotransposon families increased and spread the transcription factor binding sites

**Figure 2.**
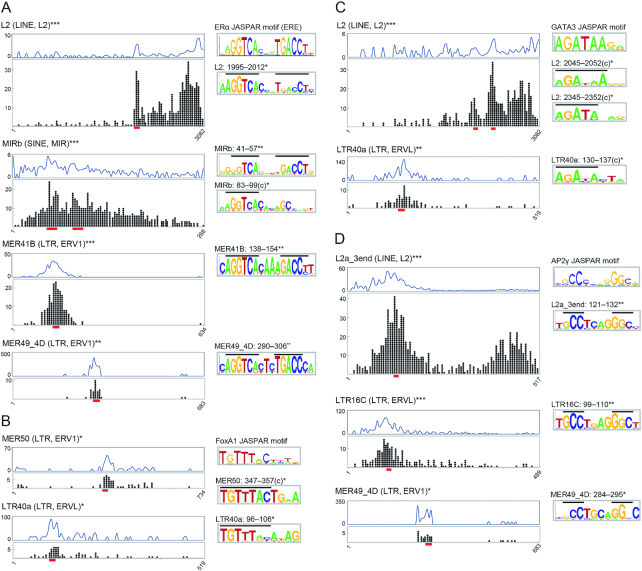
Localized distribution of the transcription factor binding sites and presence of binding motifs in TEs. (A–D) Representative TE subfamilies associated with ERα (**A**), FoxA1 (**B**), GATA3 (**C**) and AP2γ (**D**). Additional examples are shown in Supplementary Figures S4 and S5. Dot plots represent all binding events where the positions correspond to the TE consensus sequence (*x* axis). Proportions of the number of binding events (10^−5^) among all TE copies in the human genome (i.e. the normalized distribution of binding sites) are shown above the dot plots. Asterisks to the right of the TE names indicate the significantly non-uniform distribution of the binding sites within the consensus sequences (two-tailed Fisher's exact test and χ^2^-test for *n* ≤ 100 and *n* > 100, respectively; *P < 0.05, **P < 10^–5^, ***P < 10^–10^). In the binding peak regions (red lines), binding motifs were found in the TE sequences (sequence logos on the right) as shown in Supplementary Figure S10. Positions of the binding sites (c, reverse-complement) and the significant presence of the motif in the TE consensus sequences are shown above the logos (*P < 0.05, **P < 10^–5^; FIMO analysis (34)). Horizontal lines in logos represent conserved nucleotides shared with the known JASPAR motifs shown in the upper right of each panel.

Many TEs including the 55 families identified above may possess binding motifs for the four transcription factors analyzed here. To demonstrate this, the genomic position of each of the 38 500 TE-associated binding sites was converted to a site position corresponding to the consensus sequence of each TE subfamily (Figure 2). Ancient L2 and MIR elements had localized binding sites for ERα, where the known binding motifs exist in the TE consensus sequences (Figure 2A). Likewise, MER41, which is known to have STAT1 binding sites (18), and MER49_4D, an ERV1 family newly characterized here, also possess these palindromic binding motifs (Figure 2A). Furthermore, I found strong indication that at least thousands of FoxA1, GATA3, and AP2γ binding sites result from amplification of the binding motifs by retrotransposons such as L2, MER50, LTR40, LTR16 and MER49_4D (Figure 2B–D, Supplementary Figures S9 and S10). Because in general many copies of some kinds of TEs, such as LINEs, are 5′-truncated (55), the proportion of the number of binding events among the number of copies per site was calculated to normalize the difference in the length of TEs. This normalization resulted in similar distributions (blue graphs in Figure 2, Supplementary Figures S4 and S5) with only a few exceptions, such as L2a_3end bound by AP2γ in which one of the two dot-plot peaks in the 450-bp region showed no strong enrichment after normalization (Figure 2D). Overall, in this study, a total of 13, 13, 7 and 16 kinds of TE subfamilies, with or without enrichment (Supplementary Figure S3), showed explicit binding peaks for ERα, FoxA1, GATA3 and AP2γ, respectively, wherein their binding motifs are present in the consensus (Figure 2, Supplementary Figures S4, S5 and S10–S14). Notably, the L2 family possessed binding motifs for all four factors (Figure 2, Supplementary Figures S4 and S9), suggesting its possible greater impact on the organization of the *cis*-regulatory network during evolution.

The enhancer function of an ERα-binding L2 sequence was tested (Supplementary Figure S15). The ERα-binding site, corresponding to basepairs 1995–2012 of the L2 consensus sequence (Figure 2A), is conserved among eutherians and is located 68 kb upstream of the nearest gene, *Igf1* (Supplementary Figure S15A–C), which is essential for mammary gland branching morphogenesis (56). The human, marmoset, and elephant sequences all showed enhancer activity in luciferase reporter assays in MCF-7 cells, and disruption of the binding site resulted in diminished activity (Supplementary Figure S15D). Because the corresponding insertion of the L2 was not observed in the orthologous regions of marsupials (opossum and wallaby) and platypus (data not shown), this result suggests that the L2 element might have been co-opted/exapted in a eutherian ancestor. Therefore, some portion of the ERα-binding TEs may potentially act as developmental enhancers, although further validation will be necessary to understand in detail their mechanisms and roles, such as epigenetic states, timing, and enhancer-gene interactions.

### Two-phased acquisition of the ERα-binding TEs during mammalian evolution

**Figure 3.**
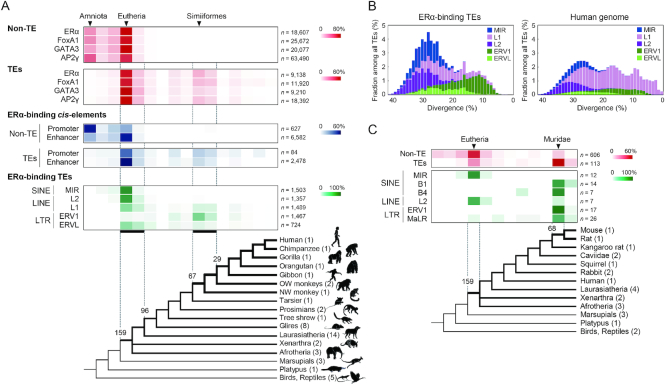
Estimated time of acquisition of the transcription factor binding sequences during mammalian evolution. (**A**) Heat maps represent the proportion of the number of binding sites for each transcription factor as compared among the clades wherein the orthologs are distributed. The ERα-binding sites were mapped separately among the promoters and enhancers (blue), as estimated by ChromHMM (Supplementary Figure S2), or among the top five highest contributing TE categories, MIR, L2, L1, ERV1 and ERVL (green) (see also Supplementary Figure S8). Values above the tree represent divergence times (in million years ago, Mya). The number of species analyzed is shown after the clade names. OW monkeys, Old World monkeys; NW monkey, New World monkey. (**B**) Distribution of the top five contributing TE groups according to the divergence (%) from the consensus sequence compared among ERα-binding TEs (left) and all TE copies (right) in the human genome. Note that the *x* axis is inverted to show old and young copies on the left and right, respectively. (**C**) Proportion of the number of ERα-binding sites in the mouse mammary gland as compared among the clades wherein the orthologs are distributed. The TE-associated binding sites (red) were separately mapped among the top six highest contributing TE categories, MIR, B1, B4, L2, ERV1 and MaLR (green).

To estimate the timing for the origin of the binding sites during evolution, the presence/absence of the orthologous sequences of the binding sites were explored among amniotes. For non-TE binding sites, over 95% of them originated in a eutherian ancestor or at an earlier time (Figure 3A). In contrast, the origins of 59–72% of the TE-associated binding sites were traced back to the common ancestor of Eutheria or Boreoeutheria (159–96 Mya), and, additionally, 13–22% were acquired before or after divergence of New World monkeys (67–29 Mya). The ERα-binding non-TE promoters existed primarily in an amniote ancestor, and, subsequently, enhancers were gained by the time of a eutherian ancestor (Figure 3A, blue), whereas ERα-binding TEs showed a similar distribution between promoters and enhancers, i.e., representing a two-phased acquisition. Partitioning of the ERα-binding TEs into TE categories revealed that the gain in the earlier phase resulted primarily from MIR and L2 (49.1% of all TEs), whereas ERV1 showed a maximum contribution to the later phase (49.0%; Figure 3A, green). This conclusion is supported by an age distribution of the ERα-binding TEs representing a large contribution of L2 and MIR in an older era and a maximum contribution of ERV1 in relatively recent times (Figure 3B).

The massive gains in the potential binding sites from TEs might have led to the expression of various genes during mammary gland morphogenesis. To address this, an ontology analysis was conducted with GREAT (52) for the neighboring genes of the TE-derived active promoters/enhancers bound by each of the four factors and p300. This analysis revealed a significant enrichment for dozens of biological functions that include mammary gland morphogenesis and estrogen response (Supplementary Figure S6), suggesting that some of the thousands of TEs may serve as enhancers of genes involved in mammary gland development.

Furthermore, a chromosomal distribution analysis of TEs bound by the four factors revealed that a human 3.7-Mb locus (chr20:45 820 001–49 520 000; hg19) showed the highest TE density (Supplementary Figure S7A, B). This locus contains 24 protein-coding genes (Supplementary Figure S7C), among which at least 10 (e.g. *NCOA3*, *SNAI1*/*Snail1*, *CEBPB* and *PTPN1*) are involved in mammary gland development, ERα-related regulation, or breast cancer (Supplementary Table S1), suggesting an importance of this locus for mammary gland morphogenesis. This chromosomal locus partly overlaps with one of the frequently amplified regions in breast cancers, whereas other known amplified regions in MCF-7 cells (57) showed no such signal (Supplementary Figure S7), suggesting a possible involvement of TEs in this locus in the regulation of breast cancer cell proliferation.

### The evolutionary landscape of the ERα-binding sites in mouse

To compare the evolutionary landscape of ERα-binding sites between human and mouse, ChIP-seq data for ERα in the mouse mammary gland tissue (49) were similarly analyzed. Among a total of 774 ERα-binding sites, 113 (14.6%) were identified to originate from TEs although the total number of binding sites was small compared with the results in the human genome (Figure 1B) presumably because of a difference in the origins of samples used for ChIP-seq experiments (cell culture and tissue). The proportion of TEs among the binding sites is smaller than that in the mouse genome (41%), whereas the proportion is higher than that in putative functional elements such as CDSs and CNEs in the mouse (0.3% and 5.6%, respectively; Supplementary Figure S16A). In a eutherian ancestor, the binding sites resulted from mainly L2 and MIR, whereas in a murine ancestor, ERV1 and MaLR families as well as B1 and B4 SINEs have provided over half of them (64 sites, 56.6%) (Figure 3C). One of the ERα-binding L2 elements in the mouse was the ortholog of the human L2 locus used in the reporter assay (Supplementary Figure S15), suggesting a conserved function of the locus among mammals. Remarkably, RLTR14_RN, a Muridae-specific ERV1 family occupying only 0.01% of the mouse genome, constitute 10.6% (12/113) of the ERα-binding TEs (134-fold enrichment, *P* < 10^–16^, two-tailed binomial test). The ERα-binding motif found in basepairs 136–150 of the RLTR14_RN consensus sequence is conserved among the copies (Supplementary Figure S16B). Therefore, RLTR14_RN is one of the highest contributing TEs by which potential ERα-binding sites have been spread in the murine lineage. Thus, both humans and mice showed a similar two-phased acquisition of TE-derived ERα-binding sites during evolution (Figure 3).

## DISCUSSION

### Wave of L2/MIR exaptation in a eutherian ancestor

The two-phase expansion of retrotransposons consisting mainly of L2/MIR and ERV1 provided a vast number of potential sources of *cis*-elements, which might then have had a substantial impact on the establishment and modification, respectively, of the gene regulatory network for mammary gland development. Both MIR and its retropositional partner L2 are ancient TEs. The L2 clade LINEs are distributed widely in vertebrates, and the origin of MIR can be traced back at least to the last common ancestor of amniotes (312 Mya) (58). Given this fact as well as their high divergence from the consensus sequence (Figure 3B), the retrotranspositional activity of the L2/MIR elements is considered to have been retained until the common ancestor of the eutherians (55). Because most of the ancient L2/MIR copies represent a high sequence divergence, in this study the ChIP-seq reads were not directly compared to the TE consensus sequences to avoid the possibility that differences in the divergence among TEs could affect identification of the TE-associated binding sites.

In general, a number of nucleotide substitutions have accumulated in TE sequences. The vast majority (>99%) of ancient TEs have not been under sustained purifying selection (5). In a recent work, targeted deletion of a large cluster of L1 retrotransposons in mice had no impact on local transcriptional control (59). These findings indicate that most newly inserted TE sequences are under neutral evolution and have no beneficial function in the host genome (2). Contrary to the traditional view, presumably long after the retrotransposition, in a eutherian ancestor, utilization of a part of the L2/MIR-derived ‘seed’ binding sites for the transcriptional regulators might have had evolutionary benefits in terms of *cis*-regulatory improvement for mammary gland development. Eutherians indeed have different morphogenetic features of their mammary glands as compared with those of marsupials/monotremes. For example, most eutherians produce multiple primary sprouts per mammary bulb, whereas monotremes have a plate-like mammary bulb and marsupials have a single primary sprout per mammary gland (60). Aspects of these eutherian-specific characteristics might have resulted from co-options/exaptations of the ancient L2 and MIR elements (Figure 3A). Once the TE sequences were co-opted and were used as distal enhancers, some of the binding sites would have evolved under purifying selection, as shown by the existence, to some extent, of inter-species conservation in the binding sites on TEs (Figure 1C, Supplementary Figures S17–S20). In addition, inter-copy comparisons of the TEs showed higher sequence similarity in the binding sites (as represented by blue and light blue in Supplementary Figure S10), whereas relatively more sequence substitutions accumulated in non-binding site regions, which is clearly observed in ancient TEs such as L2 and MIR (Supplementary Figure S10A–C, H, I, K, L). The sequence conservation implies the presence of purifying selection, suggesting that the binding sites have some functions. In the present study, because the TE-derived binding sites were identified in MCF-7, which is a breast cancer cell line, it is reasonable to question whether the TEs are also functional in normal breast cells. However, it should be noted that the signatures of purifying selection described above were found in the human reference genome (hg19). Although experimental assays using the normal cells will be necessary in the future, this fact suggests that the functions of the binding sites in normal cells have been retained during the evolution of the lineage leading to humans.

It is a remarkable finding that L2 increased a number of potential binding modules for all four factors (ERα, FoxA1, GATA3 and AP2γ) involved in mammary gland development (Figure 2, Supplementary Figures S4 and S9). LINEs could possess a variety of potential binding motifs because of their length (3–6 kbp) and high copy number (∼10^5^), although SINEs and LTRs are the TEs that have most frequently been reported to have been co-opted during evolution (7–11). It is possible that the ancient LINE family might have been co-opted/exapted during another period of time and might thus have provided binding sites for other transcription factors. Future studies may reveal further cases of exaptation of LINEs involved in morphological evolution.

Among the TEs, the MIR SINE family provided the most ERα binding sites (Figures 2 and 3A) (12,54). A recent study revealed that MIRs also contain a binding site for ZNF768, and indeed >10^4^ copies are bound by this transcription factor in human cells (61). These findings raise the possibility that MIR as well as L2 might have contributed substantially to the expansion of binding sites for several other kinds of transcription factors, which could have led to developmental changes during mammalian evolution.

One of the most remarkable characteristics of MIR is that this SINE family belongs to the CORE-SINE superfamily. Over 10 SINE members of this superfamily share a highly conserved ∼65-bp ‘CORE’ sequence in the central region, but its role is unclear (62–64). It should be noted that the latter of the two ERα-binding sites and the ZNF768-binding sequence referred to above (61) are located within the CORE region of MIR (Figure 2A, Supplementary Figure S9). If the CORE sequence contains binding motifs of pivotal transcription factors, it is possible that the CORE-SINE members confer an advantage to the host species. Furthermore, other SINE superfamilies such as V-SINEs (65), DeuSINEs (8) and MetaSINEs (66) are widely distributed in animals. For example, hundreds of copies of the AmnSINE1 family, a member of the DeuSINEs, are highly conserved evolutionarily, and a subset of them indeed has enhancer properties (8,10,11,67). Therefore, it is important to fully understand which transcription factors can bind to the central sequences of the SINE superfamily members, including the CORE of MIR, to reveal their beneficial effects on the host.

### Lineage-specific exaptation of ERVs

Meanwhile, in primates, ERV1s might have participated in simian-specific changes in mammary gland development, although determining detailed organogenetic differences between simians and non-simian primates remain an issue. Unexpectedly, in Muridae, ERV elements have independently provided ERα binding sites (Figure 3C, Supplementary Figure S16B). Because humans and mice have some differences in mammary gland development (68), the ERα-associated ERVs might have provided a *cis*-regulatory difference for the lineage-specific features of the mammary gland. In general, a variety of different ERV families are distributed in each mammalian clade at the order/family level. For example, among all 303 subfamilies of ERV1 known in the human genome, 94 (31%), 90 (30%) and 39 (13%) arose in the common ancestor of primates (74–90 Mya), simians (43–67 Mya) and catarrhines (29–43 Mya), respectively (data not shown). Because some kinds of ERVs are known to have contributed to lineage-specific *cis*-regulatory alteration (13–15,18,19), many other ERVs may also have an accelerating effect on lineage-specific modification of gene regulatory networks during evolution.

### TEs as potential drivers of *cis*-regulatory evolution

Britten and Davidson proposed that many repetitive sequences are sources of functional elements (69). Recent studies supported this model by demonstrating that several kinds of TEs have spread a variety of *cis*-regulatory modules (12–19), e.g. spreading of the binding sites of CTCF by three mammalian SINE families (B2, SINEC, and MAR1) (70), of OCT4 by MER74A (13) and of STAT1 by human MER41 and rodent RLTR30B (18). In the present study, no fewer than a total of 18 TE families (composed of 26 subfamilies), of which 15 are retrotransposons, were identified as having consensus sequences that contain the potential binding motifs, and indeed the binding events are significantly enriched in the TEs and are more or less enriched even at the positions of the potential binding motifs in the consensus (Figure 2, Supplementary Figures S3 and S4). Furthermore, Britten and Davidson also proposed that repetitive elements could contribute to alteration of gene regulatory systems leading to developmental modification (69). However, it has been a long-standing question whether retrotransposons accelerated an increase in transcription factor binding sites involved in common mammalian morphological features during evolution. Here, by providing a strong indication that retrotransposons have facilitated an increase in enhancers bound by the four transcription factors involved in mammary gland development, this study shed light on the deep involvement of TEs in gene regulatory evolution for common morphological features in mammals.

### Potential evolutionary benefits of TEs to the host

Over 4.5 million copies of TEs occupy the human genome. Despite recent efforts to uncover many co-opted/exapted TEs, it is still largely unknown how many of them have beneficial functions. In the present study, 38 500 copies of human TEs were revealed to possess binding sites for at least one of the four transcription factors. Future studies may uncover hundreds of thousands of TE copies harboring binding sites for many other transcription factors.

In addition to their contributions of *cis*-regulatory elements, many TEs were exapted as various functional modules such as protein-coding exons (71) and boundaries of chromatin domains (70,72). For example, >100 copies of ancient MIRs act as a part of protein-coding exons, which arose >100 Mya (71), a similar timing to the co-option of L2/MIRs in this study (Figure 3A). In addition, many genes are known to be derived from LTR-retrotransposons. For example, mammalian syncytin genes that are required for normal placental development are derived from ERVs, and these exaptations occurred several times independently in diverse lineages (73). In primates, two syncytin genes emerged before and after the divergence of New World monkeys (74), which is a similar period of time to the expansion of the ERV1-derived binding sites for the four transcription factors analyzed here (Figure 3A). Therefore, some TEs such as ERVs had significant evolutionary effects on the expansion of lineage-specific functional sources in mammals, as discussed previously (14,15,18).

Taken together, the results of this study suggests that dozens of TE families increased and spread the binding sites for four transcription factors, which could lead to a dramatic increase of their downstream genes involved in mammary gland development. In addition to mammary glands, mammals share many features such as the neocortex, closed secondary palate, hair, etc. Thus, future research should uncover many additional kinds of TEs similarly involved in the morphogenesis of these features in mammals. Given the findings in this study and the fact that vertebrate genomes contain a huge number of TE copies (1), co-option/exaptation of retrotransposons as potential sources of *cis*-elements for pivotal developmental transcription factors may be, in general, one of the facilitators of large-scale morphological innovations during evolution.

## Supplementary Material

gkz1003_Supplemental_File
