# Supplementary material for: Retrotransposons spread potential cis-regulatory elements during mammary gland evolution
Source: Nucleic Acids Res. 2019 Oct 23;47(22):11551–62. doi: 10.1093/nar/gkz1003 (PMC7145552; doi:10.1093/nar/gkz1003)
Supplement: gkz1003_Supplemental_File [file gkz1003_supplemental_file.pdf]

Supplementary Material for "Retrotransposons spread potential *cis*-regulatory elements during mammary gland evolution"

Hidenori Nishihara

Table of contents

Figure S1	Page 2
Figure S2	Page 3
Figure S3	Pages 4–5
Figure S4	Pages 6–7
Figure S5	Pages 8–9
Figure S6	Pages 10–11
Figure S7	Page 12
Figure S8	Page 13
Figure S9	Page 14
Figure S10	Pages 15–19
Figure S11	Pages 20–21
Figure S12	Pages 22–25
Figure S13	Page 26
Figure S14	Pages 27–30
Figure S15	Page 31
Figure S16	Page 32
Figure S17	Page 33
Figure S18	Page 34
Figure S19	Page 35
Figure S20	Page 36
Table S1	Page 37
Table S2	Page 38
Supplementary References	Pages 39–40

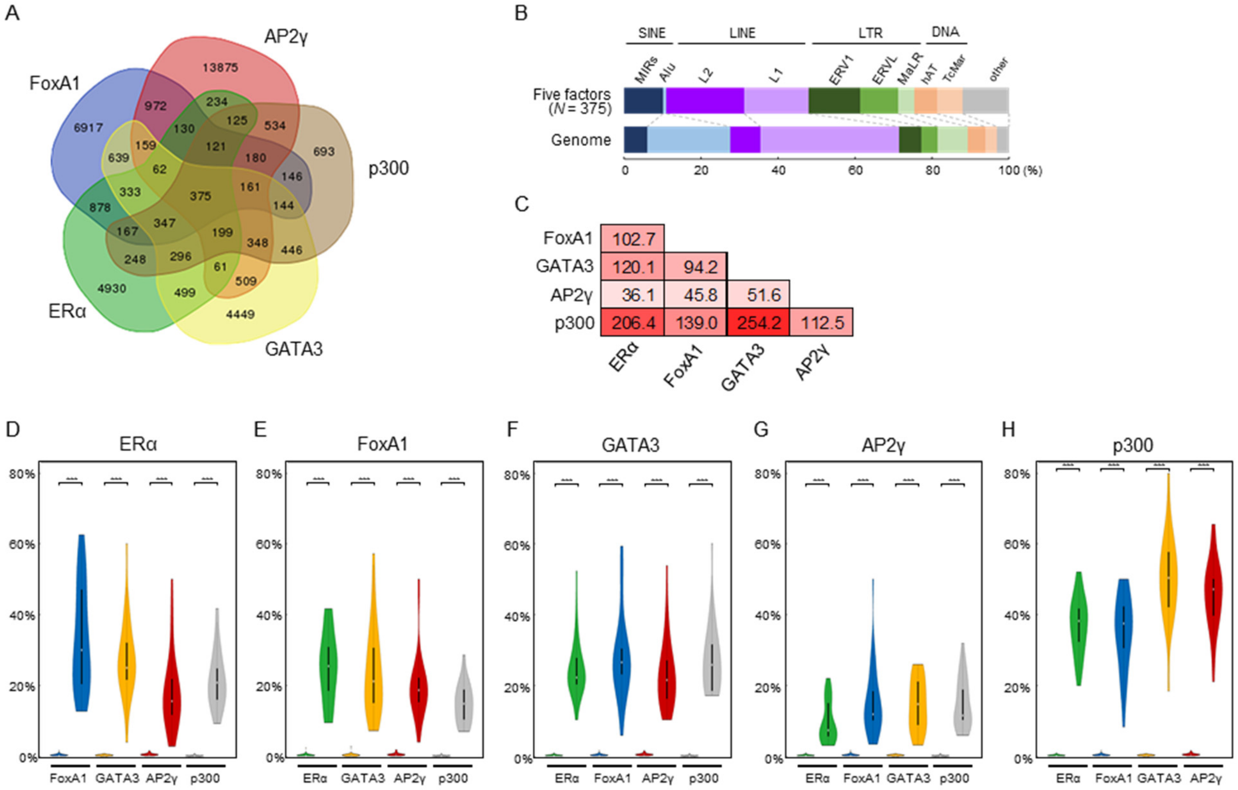

Figure S1. Enrichment of multiple transcription factor binding sites on the same TE sequence. (**A**) Venn diagram showing the number of TE copies containing binding sites of all combinations of the four transcription factors and a co-activating factor p300. (**B**) Relative proportion of the TE categories bound by all five factors shown in (**A**) compared with the proportion of all human TEs (genome). (**C**) Fold enrichment of binding sites located in the same TE sequence for all combinations of the two factors. (**D–H**) Violin plots representing the proportion of the binding events for the transcription factors shown below each graph under the condition of binding of ERα (**D**), FoxA1 (**E**), GATA3 (**F**), AP2γ (**G**), and p300 (**H**) within the same TE copy. The number of binding events was calculated separately for each TE superfamily/clade for the plots. For each combination, expected and observed proportions are shown on the left and right, respectively. All combinations showed a significant enrichment (χ^2-test, $***P < 10^{-10}$).

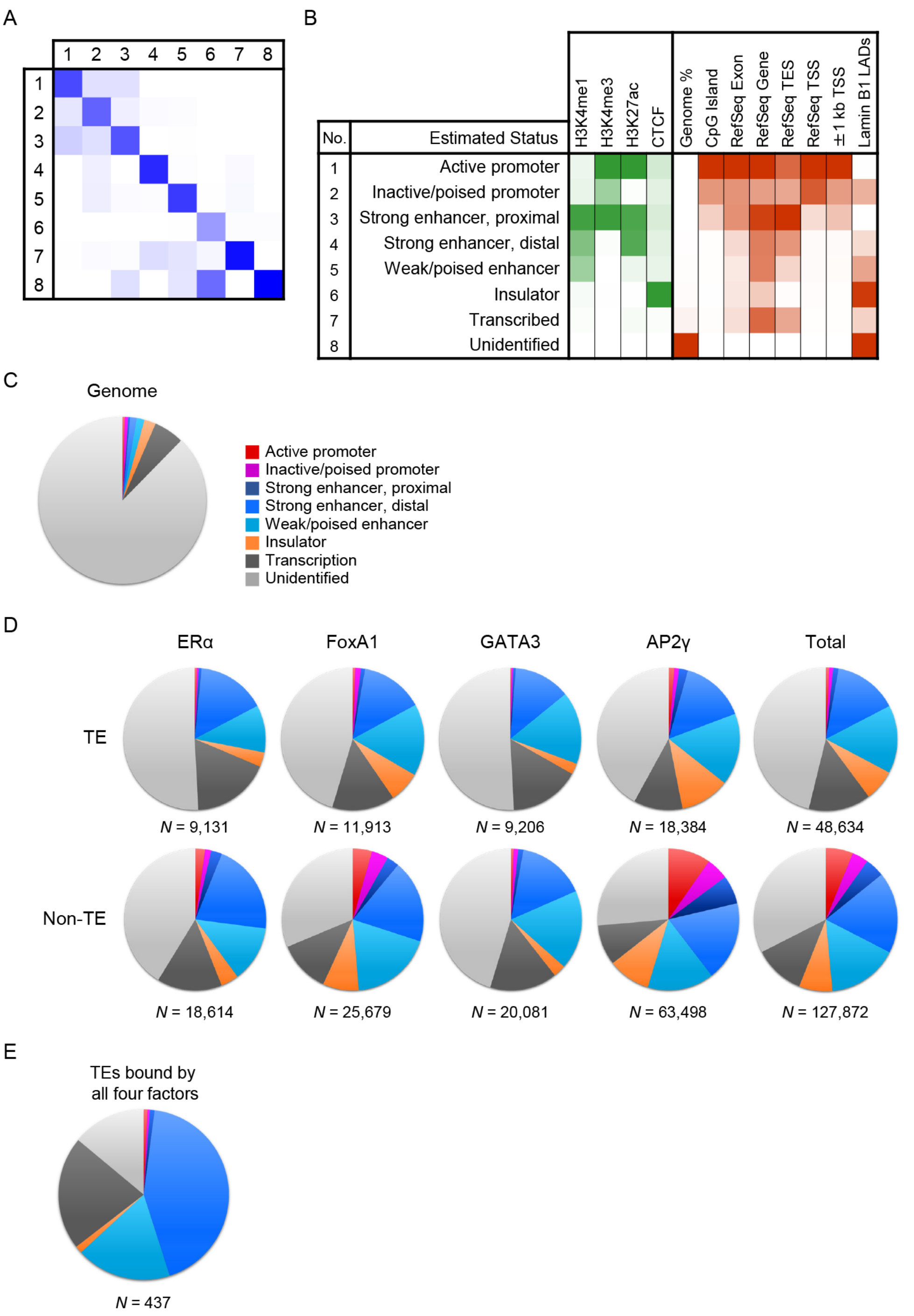

Figure S2. Functional annotation of the MCF-7 genome. (**A**) Distribution of transition parameters among the eight states produced by ChromHMM (1). (**B**) Classification of the eight states shown in panel (**A**). The functions are estimated based on the emission probability matrix for the histone marks (H3K4me1, H3K4me3, and H3K27ac) and CTCF binding (left) as well as the enrichment in various genomic annotations (right) produced by ChromHMM. (**C**) Proportion of the estimated functional regions in the whole genome. (**D**) Proportion of the estimated function of the TE (top) and non-TE (bottom) binding sites for ERα, FoxA1, GATA3, and AP2γ. (**E**) Proportion of the estimated function of non-redundant TE copies bound by all four transcription factors as revealed in Figure S1A.

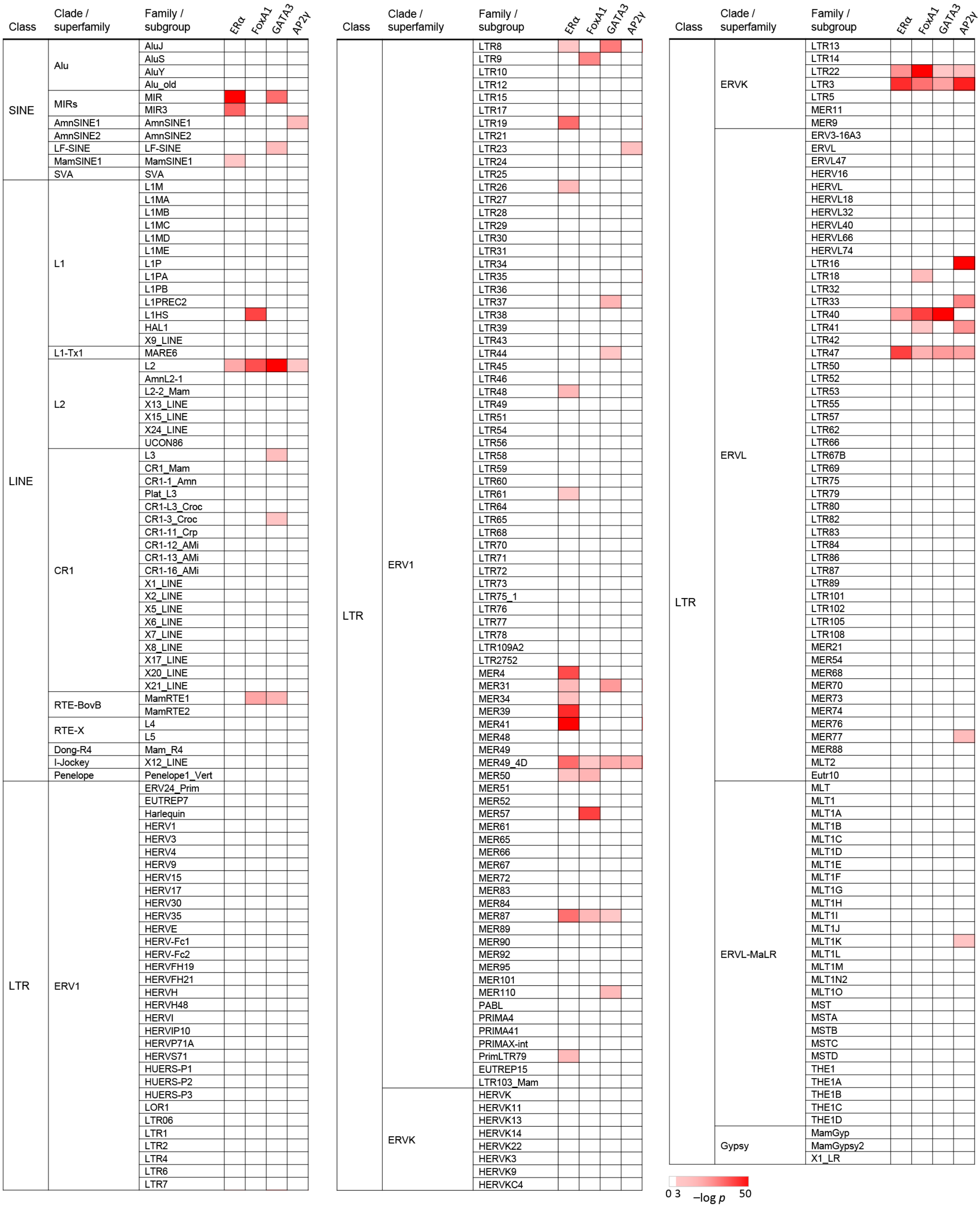
Class
Clade / superfamily
Family / subgroup
ERα
FoxA1
GATA3
AP2γ
SINE
Alu
AluJ
AluS
AluY
Alu_old
MIRs
MIR
MIR3
AmnSINE1
AmnSINE1
AmnSINE2
AmnSINE2
LF-SINE
LF-SINE
MamSINE1
MamSINE1
SVA
SVA
LINE
L1
L1M
L1MA
L1MB
L1MC
L1MD
L1ME
L1P
L1PA
L1PB
L1PREC2
L1HS
HAL1
X9_LINE
L1-Tx1
MARE6
L2
L2
AmnL2-1
L2-2_Mam
X13_LINE
X15_LINE
X24_LINE
UCON86
CR1
L3
CR1_Mam
CR1-1_Amn
Plat_L3
CR1-L3_Croc
CR1-3_Croc
CR1-11_Crp
CR1-12_AMi
CR1-13_AMi
CR1-16_AMi
X1_LINE
X2_LINE
X5_LINE
X6_LINE
X7_LINE
X8_LINE
X17_LINE
X20_LINE
X21_LINE
RTE-BovB
MamRTE1
MamRTE2
RTE-X
L4
L5
Dong-R4
Mam_R4
I-Jockey
X12_LINE
Penelope
Penelope1_Vert
LTR
ERV1
ERV24_Prim
EUTREP7
Harlequin
HERV1
HERV3
HERV4
HERV9
HERV15
HERV17
HERV30
HERV35
HERVE
HERV-Fc1
HERV-Fc2
HERVFH19
HERVFH21
HERVH
HERVH48
HERVI
HERVIP10
HERVP71A
HERVS71
HUERS-P1
HUERS-P2
HUERS-P3
LOR1
LTR06
LTR1
LTR2
LTR4
LTR6
LTR7
Class
Clade / superfamily
Family / subgroup
ERα
FoxA1
GATA3
AP2γ
LTR
ERV1
LTR8
LTR9
LTR10
LTR12
LTR15
LTR17
LTR19
LTR21
LTR23
LTR24
LTR25
LTR26
LTR27
LTR28
LTR29
LTR30
LTR31
LTR34
LTR35
LTR36
LTR37
LTR38
LTR39
LTR43
LTR44
LTR45
LTR46
LTR48
LTR49
LTR51
LTR54
LTR56
LTR58
LTR59
LTR60
LTR61
LTR64
LTR65
LTR68
LTR70
LTR71
LTR72
LTR73
LTR75_1
LTR76
LTR77
LTR78
LTR109A2
LTR2752
MER4
MER31
MER34
MER39
MER41
MER48
MER49
MER49_4D
MER50
MER51
MER52
MER57
MER61
MER65
MER66
MER67
MER72
MER83
MER84
MER87
MER89
MER90
MER92
MER95
MER101
MER110
PABL
PRIMA4
PRIMA41
PRIMAX-int
PrimLTR79
EUTREP15
LTR103_Mam
ERVK
HERVK
HERVK11
HERVK13
HERVK14
HERVK22
HERVK3
HERVK9
HERVKC4
Class
Clade / superfamily
Family / subgroup
ERα
FoxA1
GATA3
AP2γ
LTR
ERVK
LTR13
LTR14
LTR22
LTR3
LTR5
MER11
MER9
ERVL
ERV3-16A3
ERVL
ERVL47
HERV16
HERVL
HERVL18
HERVL32
HERVL40
HERVL66
HERVL74
LTR16
LTR18
LTR32
LTR33
LTR40
LTR41
LTR42
LTR47
LTR50
LTR52
LTR53
LTR55
LTR57
LTR62
LTR66
LTR67B
LTR69
LTR75
LTR79
LTR80
LTR82
LTR83
LTR84
LTR86
LTR87
LTR89
LTR101
LTR102
LTR105
LTR108
MER21
MER54
MER68
MER70
MER73
MER74
MER76
MER77
MER88
MLT2
Eutr10
ERVL-MaLR
MLT
MLT1
MLT1A
MLT1B
MLT1C
MLT1D
MLT1E
MLT1F
MLT1G
MLT1H
MLT1I
MLT1J
MLT1K
MLT1L
MLT1M
MLT1N2
MLT1O
MST
MSTA
MSTB
MSTC
MSTD
THE1
THE1A
THE1B
THE1C
THE1D
Gypsy
MamGyp
MamGypsy2
X1_LR
0 3
−log p
50

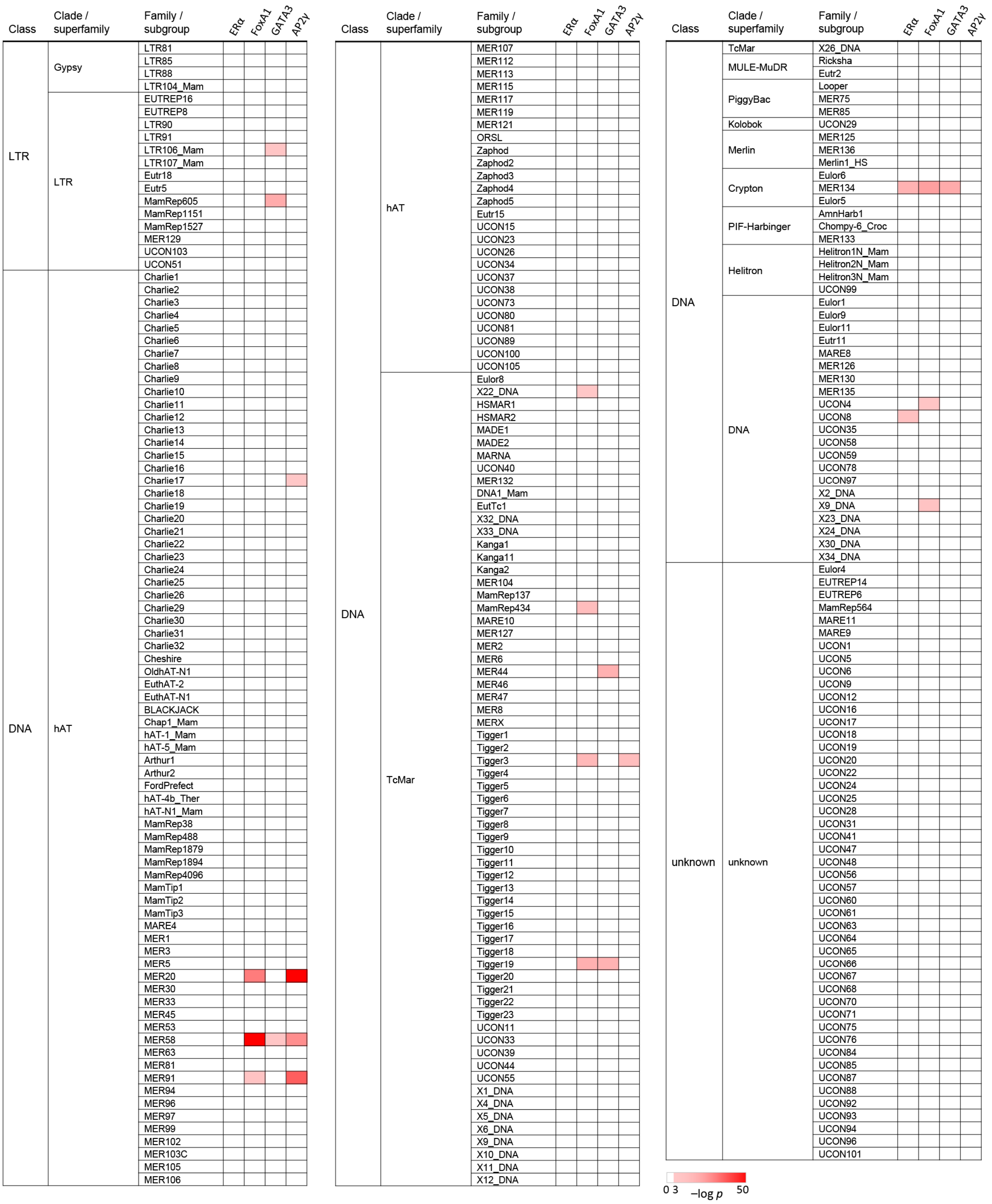

Figure S3. Enrichment of the binding events of the four transcription factors within TE families. The heat map represents the Bonferroni-corrected –log p for the 536 families of TEs bound by ERα, FoxA1, GATA3, and AP2γ (colored for $p < 0.001$, two-sided binomial test, n = 2,144).

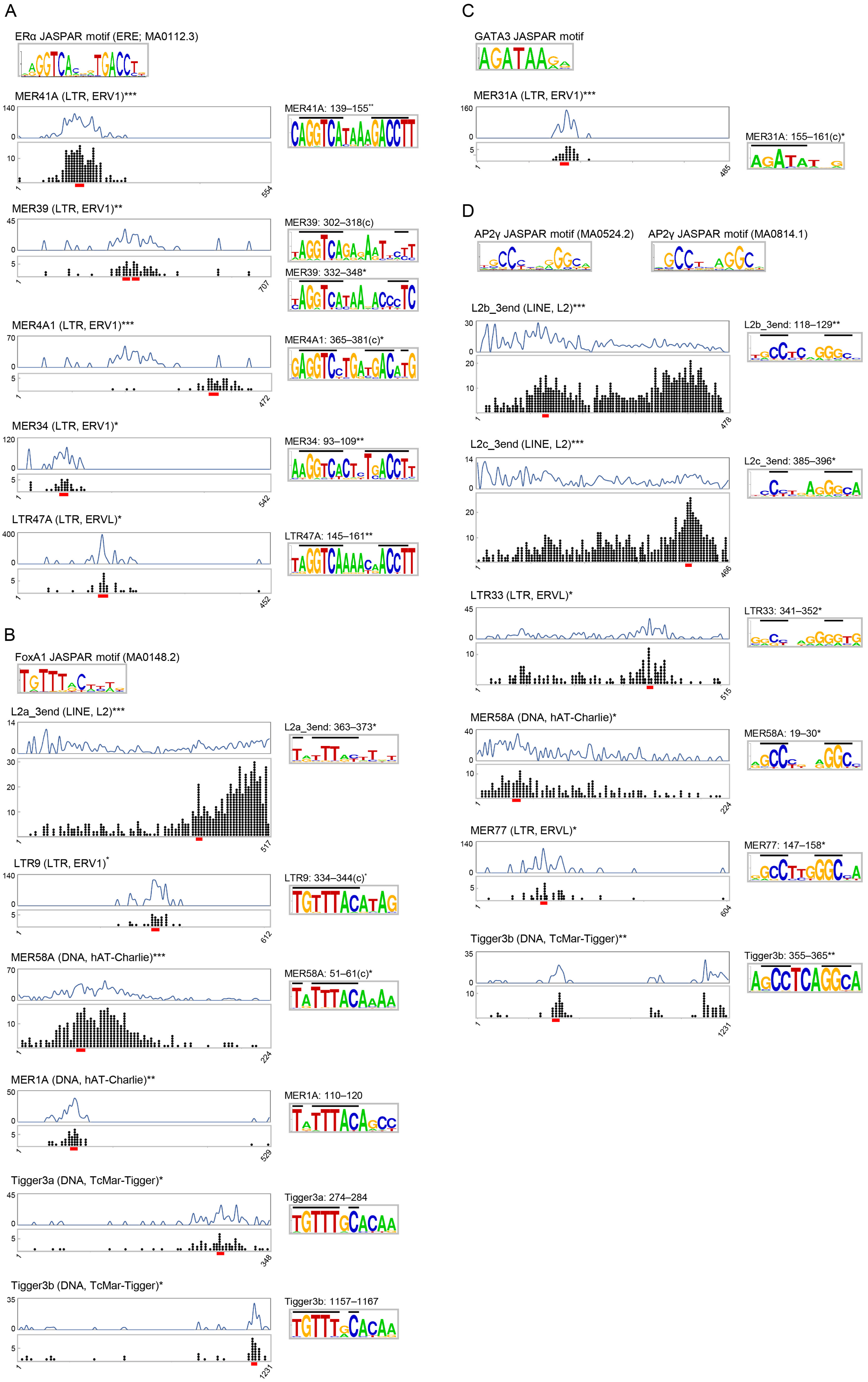
A
ERα JASPAR motif (ERE; MA0112.3)
MER41A (LTR, ERV1)***
MER41A: 139–155**
MER39 (LTR, ERV1)**
MER39: 302–318(c)
MER39: 332–348*
MER4A1 (LTR, ERV1)***
MER4A1: 365–381(c)*
MER34 (LTR, ERV1)*
MER34: 93–109**
LTR47A (LTR, ERVL)*
LTR47A: 145–161**
B
FoxA1 JASPAR motif (MA0148.2)
L2a_3end (LINE, L2)***
L2a_3end: 363–373*
LTR9 (LTR, ERV1)*
LTR9: 334–344(c)*
MER58A (DNA, hAT-Charlie)***
MER58A: 51–61(c)*
MER1A (DNA, hAT-Charlie)**
MER1A: 110–120
Tigger3a (DNA, TcMar-Tigger)*
Tigger3a: 274–284
Tigger3b (DNA, TcMar-Tigger)*
Tigger3b: 1157–1167
C
GATA3 JASPAR motif
MER31A (LTR, ERV1)***
MER31A: 155–161(c)*
D
AP2γ JASPAR motif (MA0524.2)
AP2γ JASPAR motif (MA0814.1)
L2b_3end (LINE, L2)***
L2b_3end: 118–129**
L2c_3end (LINE, L2)***
L2c_3end: 385–396*
LTR33 (LTR, ERVL)*
LTR33: 341–352*
MER58A (DNA, hAT-Charlie)*
MER58A: 19–30*
MER77 (LTR, ERVL)*
MER77: 147–158*
Tigger3b (DNA, TcMar-Tigger)**
Tigger3b: 355–365**

Figure S4. Non-uniform distribution of the transcription factor binding sites in TEs that show significant enrichment for binding events in Supplementary Figure S3. (**A–D**) Dot plots represent the binding sites of ERα (**A**), FoxA1 (**B**), GATA3 (**C**), and AP2γ (**D**) corresponding to the TE consensus sequence (*x* axis). Proportions of the number of binding events (10^{-5}) among all TE copies in the human genome (*i.e.*, normalized distribution of binding sites) are shown above the dot plots. Note that these TEs are significantly enriched among the binding sequences of the factors based on the two-sided binomial test ($p < 0.001$ with the Bonferroni correction; Supplementary Figure S3). Asterisks to the right of the TE names indicate significantly non-uniform distribution of the binding sites within the TE consensus sequences (two-tailed Fisher's exact test and $\chi 2$-test for $n \leq 100$ and $n > 100$, respectively; *$p < 0.05$, **$p < 10^{-5}$, ***$p < 10^{-10}$). In the binding peak regions (red lines), binding motifs are found in the TE sequences (sequence logos on the right). Positions of the binding sites (c, reverse-complement) and significant presence of the motif in the TE consensus sequences are shown above the logos (*$p < 0.05$, **$p < 10^{-5}$; FIMO analysis (2)). Horizontal lines in logos represent conserved nucleotides shared with the known JASPAR motifs (shown at top of panel).

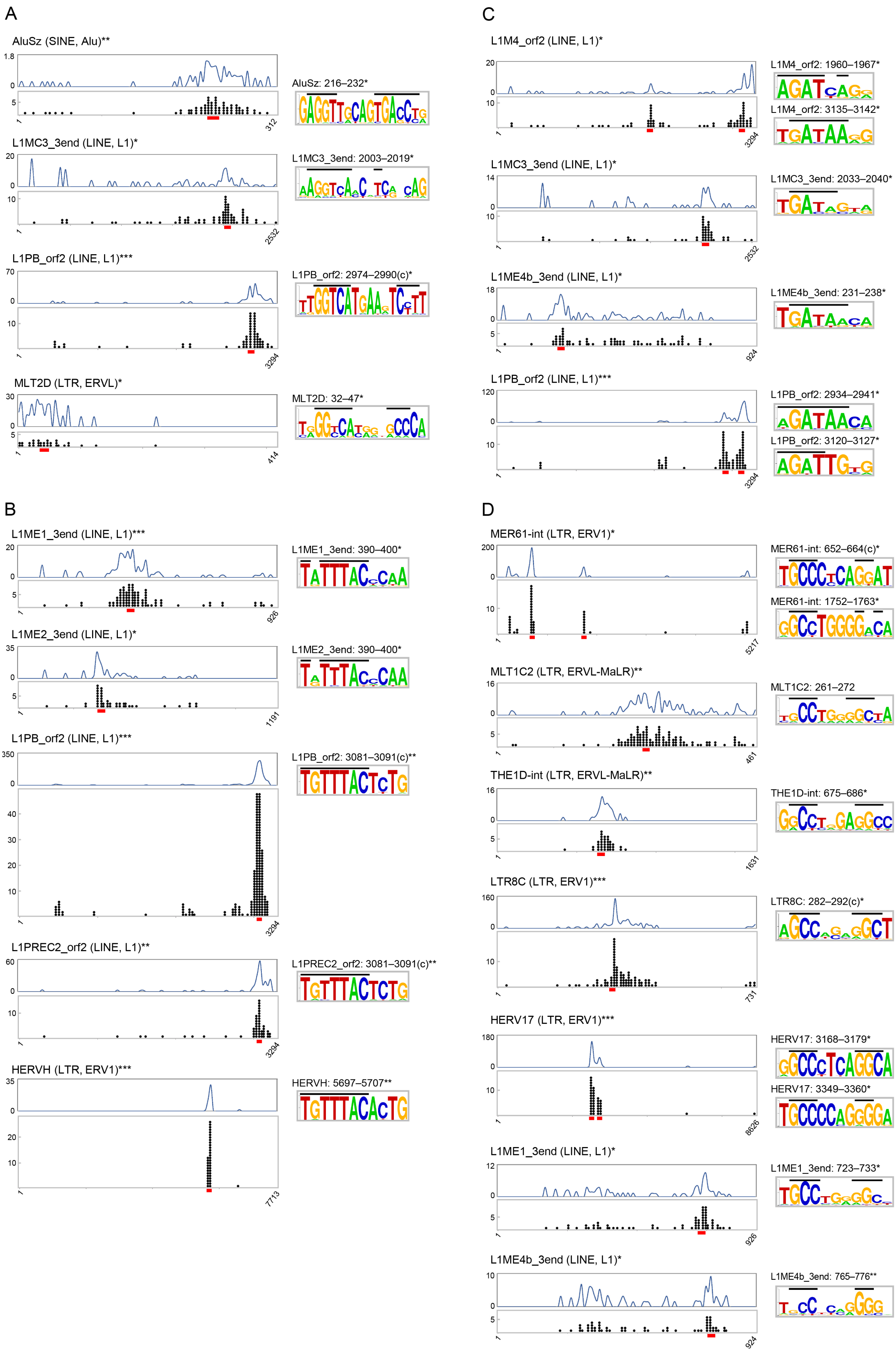
A
AluSz (SINE, Alu)**
AluSz: 216–232*
L1MC3_3end (LINE, L1)*
L1MC3_3end: 2003–2019*
L1PB_orf2 (LINE, L1)***
L1PB_orf2: 2974–2990(c)*
MLT2D (LTR, ERVL)*
MLT2D: 32–47*
B
L1ME1_3end (LINE, L1)***
L1ME1_3end: 390–400*
L1ME2_3end (LINE, L1)*
L1ME2_3end: 390–400*
L1PB_orf2 (LINE, L1)***
L1PB_orf2: 3081–3091(c)**
L1PREC2_orf2 (LINE, L1)**
L1PREC2_orf2: 3081–3091(c)**
HERVH (LTR, ERV1)***
HERVH: 5697–5707**
C
L1M4_orf2 (LINE, L1)*
L1M4_orf2: 1960–1967*
L1M4_orf2: 3135–3142*
L1MC3_3end (LINE, L1)*
L1MC3_3end: 2033–2040*
L1ME4b_3end (LINE, L1)*
L1ME4b_3end: 231–238*
L1PB_orf2 (LINE, L1)***
L1PB_orf2: 2934–2941*
L1PB_orf2: 3120–3127*
D
MER61-int (LTR, ERV1)*
MER61-int: 652–664(c)*
MER61-int: 1752–1763*
MLT1C2 (LTR, ERVL-MaLR)**
MLT1C2: 261–272
THE1D-int (LTR, ERVL-MaLR)**
THE1D-int: 675–686*
LTR8C (LTR, ERV1)***
LTR8C: 282–292(c)*
HERV17 (LTR, ERV1)***
HERV17: 3168–3179*
HERV17: 3349–3360*
L1ME1_3end (LINE, L1)*
L1ME1_3end: 723–733*
L1ME4b_3end (LINE, L1)*
L1ME4b_3end: 765–776**

Figure S5. Non-uniform distribution of the transcription factor binding sites in TEs that do not show significant enrichment for binding events in Supplementary Figure S3. (**A–D**) Dot plots represent the binding sites of ERα (**A**), FoxA1 (**B**), GATA3 (**C**), and AP2γ (**D**) corresponding to the TE consensus sequence (*x* axis), although the TEs did not show a statistically significant enrichment by the binomial test (Supplementary Figure S3). Proportions of the number of binding events (10^{-5}) among all TE copies in the human genome (*i.e.*, normalized distribution of binding sites) are shown above the dot plots. Asterisks to the right of the TE names indicate significantly non-uniform distribution of the binding sites within the TE consensus sequences (two-tailed Fisher's exact test and $\chi 2$-test for $n \leq 100$ and $n > 100$, respectively; *$p < 0.05$, **$p < 10^{-5}$, ***$p < 10^{-10}$). In the binding peak regions (red lines), binding motifs are found in the TE sequences (sequence logos on the right). Positions of the binding sites (c, reverse-complement) and significant presence of the motif in the TE consensus sequences are shown above the logos (*$p < 0.05$, **$p < 10^{-5}$; FIMO analysis (2)). Horizontal lines in logos represent conserved nucleotides shared with the known JASPAR motifs (shown at top of panel).

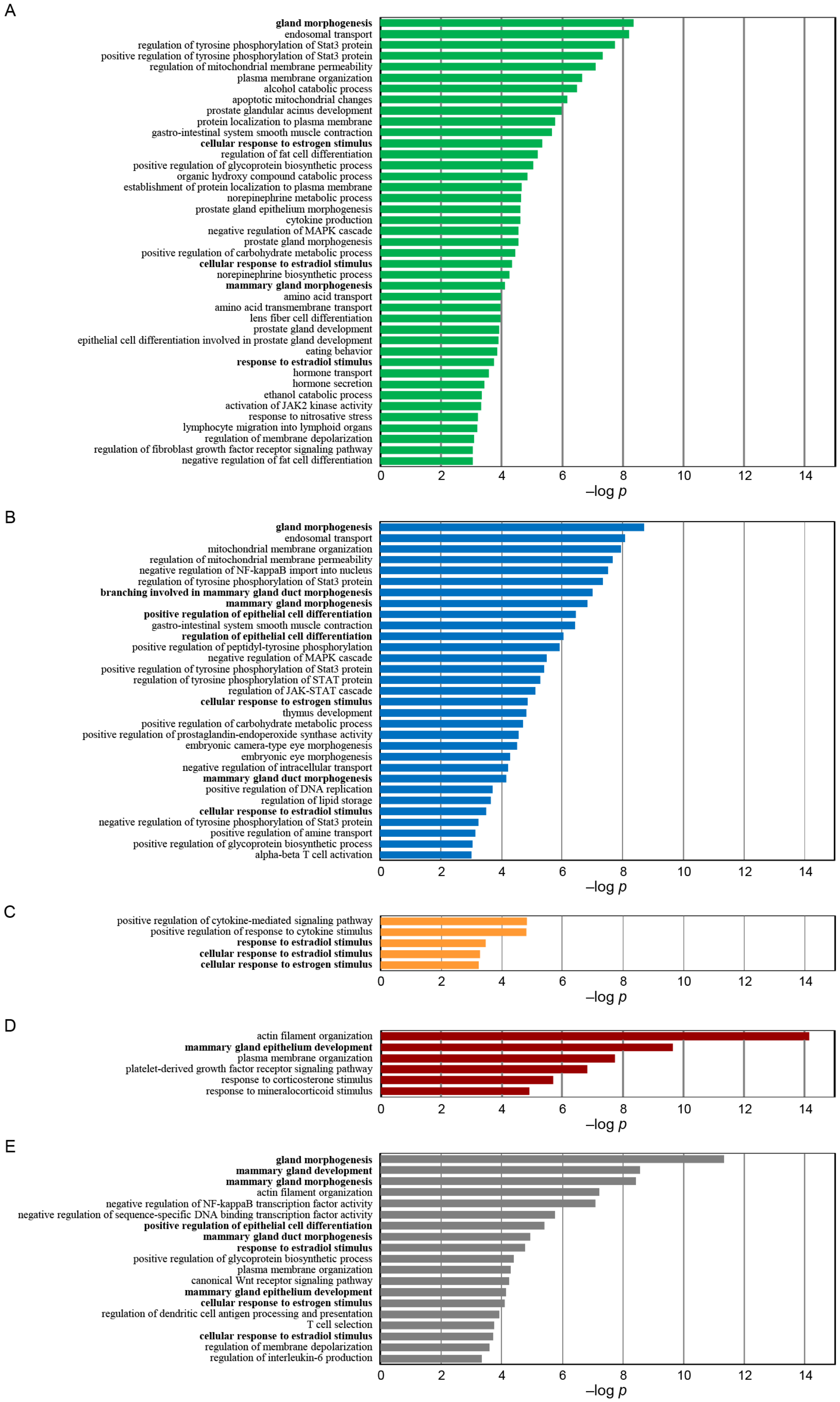
A
gland morphogenesis
endosomal transport
regulation of tyrosine phosphorylation of Stat3 protein
positive regulation of tyrosine phosphorylation of Stat3 protein
regulation of mitochondrial membrane permeability
plasma membrane organization
alcohol catabolic process
apoptotic mitochondrial changes
prostate glandular acinus development
protein localization to plasma membrane
gastro-intestinal system smooth muscle contraction
cellular response to estrogen stimulus
regulation of fat cell differentiation
positive regulation of glycoprotein biosynthetic process
organic hydroxy compound catabolic process
establishment of protein localization to plasma membrane
norepinephrine metabolic process
prostate gland epithelium morphogenesis
cytokine production
negative regulation of MAPK cascade
prostate gland morphogenesis
positive regulation of carbohydrate metabolic process
cellular response to estradiol stimulus
norepinephrine biosynthetic process
mammary gland morphogenesis
amino acid transport
amino acid transmembrane transport
lens fiber cell differentiation
prostate gland development
epithelial cell differentiation involved in prostate gland development
eating behavior
response to estradiol stimulus
hormone transport
hormone secretion
ethanol catabolic process
activation of JAK2 kinase activity
response to nitrosative stress
lymphocyte migration into lymphoid organs
regulation of membrane depolarization
regulation of fibroblast growth factor receptor signaling pathway
negative regulation of fat cell differentiation
0 2 4 6 8 10 12 14
–log p
B
gland morphogenesis
endosomal transport
mitochondrial membrane organization
regulation of mitochondrial membrane permeability
negative regulation of NF-kappaB import into nucleus
regulation of tyrosine phosphorylation of Stat3 protein
branching involved in mammary gland duct morphogenesis
mammary gland morphogenesis
positive regulation of epithelial cell differentiation
gastro-intestinal system smooth muscle contraction
regulation of epithelial cell differentiation
positive regulation of peptidyl-tyrosine phosphorylation
negative regulation of MAPK cascade
positive regulation of tyrosine phosphorylation of Stat3 protein
regulation of tyrosine phosphorylation of STAT protein
regulation of JAK-STAT cascade
cellular response to estrogen stimulus
thymus development
positive regulation of carbohydrate metabolic process
positive regulation of prostaglandin-endoperoxide synthase activity
embryonic camera-type eye morphogenesis
embryonic eye morphogenesis
negative regulation of intracellular transport
mammary gland duct morphogenesis
positive regulation of DNA replication
regulation of lipid storage
cellular response to estradiol stimulus
negative regulation of tyrosine phosphorylation of Stat3 protein
positive regulation of amine transport
positive regulation of glycoprotein biosynthetic process
alpha-beta T cell activation
0 2 4 6 8 10 12 14
–log p
C
positive regulation of cytokine-mediated signaling pathway
positive regulation of response to cytokine stimulus
response to estradiol stimulus
cellular response to estradiol stimulus
cellular response to estrogen stimulus
0 2 4 6 8 10 12 14
–log p
D
actin filament organization
mammary gland epithelium development
plasma membrane organization
platelet-derived growth factor receptor signaling pathway
response to corticosterone stimulus
response to mineralocorticoid stimulus
0 2 4 6 8 10 12 14
–log p
E
gland morphogenesis
mammary gland development
mammary gland morphogenesis
actin filament organization
negative regulation of NF-kappaB transcription factor activity
negative regulation of sequence-specific DNA binding transcription factor activity
positive regulation of epithelial cell differentiation
mammary gland duct morphogenesis
response to estradiol stimulus
positive regulation of glycoprotein biosynthetic process
plasma membrane organization
canonical Wnt receptor signaling pathway
mammary gland epithelium development
cellular response to estrogen stimulus
regulation of dendritic cell antigen processing and presentation
T cell selection
cellular response to estradiol stimulus
regulation of membrane depolarization
regulation of interleukin-6 production
0 2 4 6 8 10 12 14
–log p

Figure S6. Gene ontology analysis for the neighboring genes of the TE-derived *cis*-elements. (**A–E**) Gene ontology analysis was conducted with GREAT (3) for the genes surrounding the TEs that are estimated to function active promoters or strong enhancers (Supplementary Figure S2) bound by ERα (**A**), FoxA1 (**B**), GATA3 (**C**), AP2γ (**D**), and p300 (**E**). Biological terms significantly enriched (>2-fold enrichment, $p < 0.001$, and false discovery rate of $q < 0.05$) are shown with the –log *p*. The terms related to mammary gland development and estrogen responses are highlighted in bold.

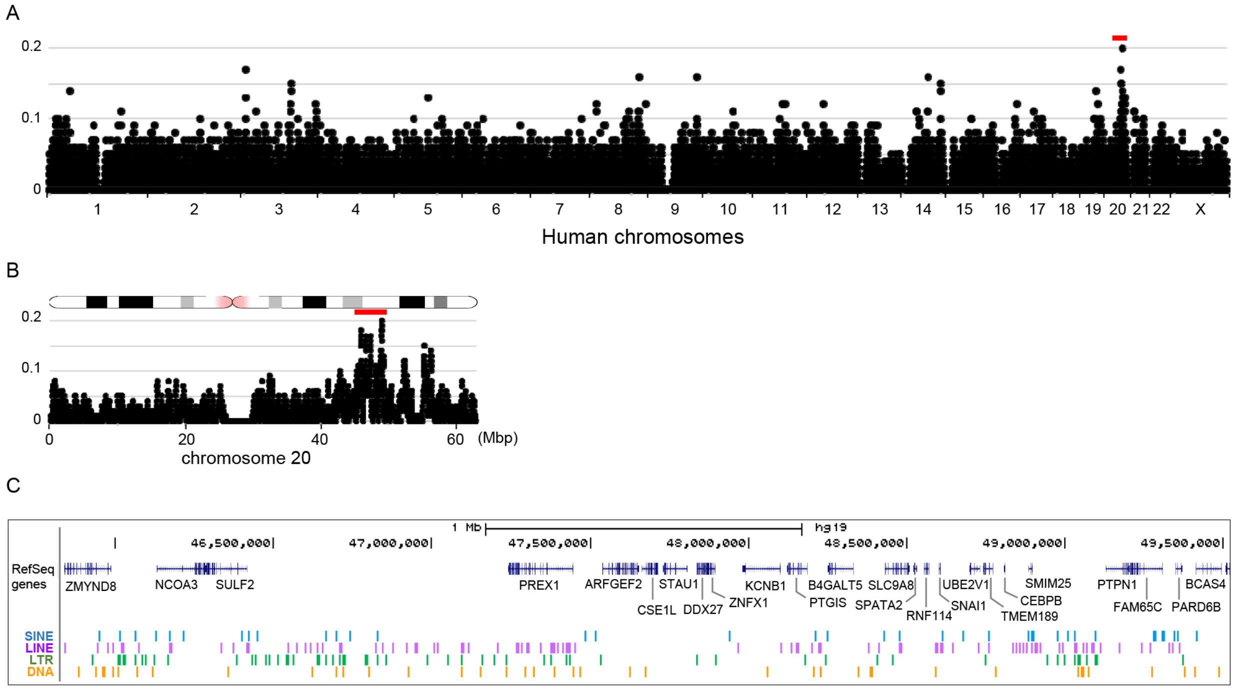

Figure S7. Density of TEs bound by these four transcription factors in the human genome. (**A, B**) Density of TEs (copies per kilobase) bound by ERα, FoxA1, GATA3, or AP2γ were calculated in sliding 100-kb windows with 50- and 10-kb steps for (**A**) the entire genome (hg19, excluding chromosome Y) and (**B**) chromosome 20. Red bars denote the region with the highest density. (**C**) RefSeq protein-coding genes and TEs in the 3.7-Mb region (chr20:45,820,001-49,520,000, hg19) showing the highest TE density in (**B**). Known functions of the 10 genes involved in mammary gland development, ERα-related regulation, or breast cancer are listed in Supplementary Table S1.

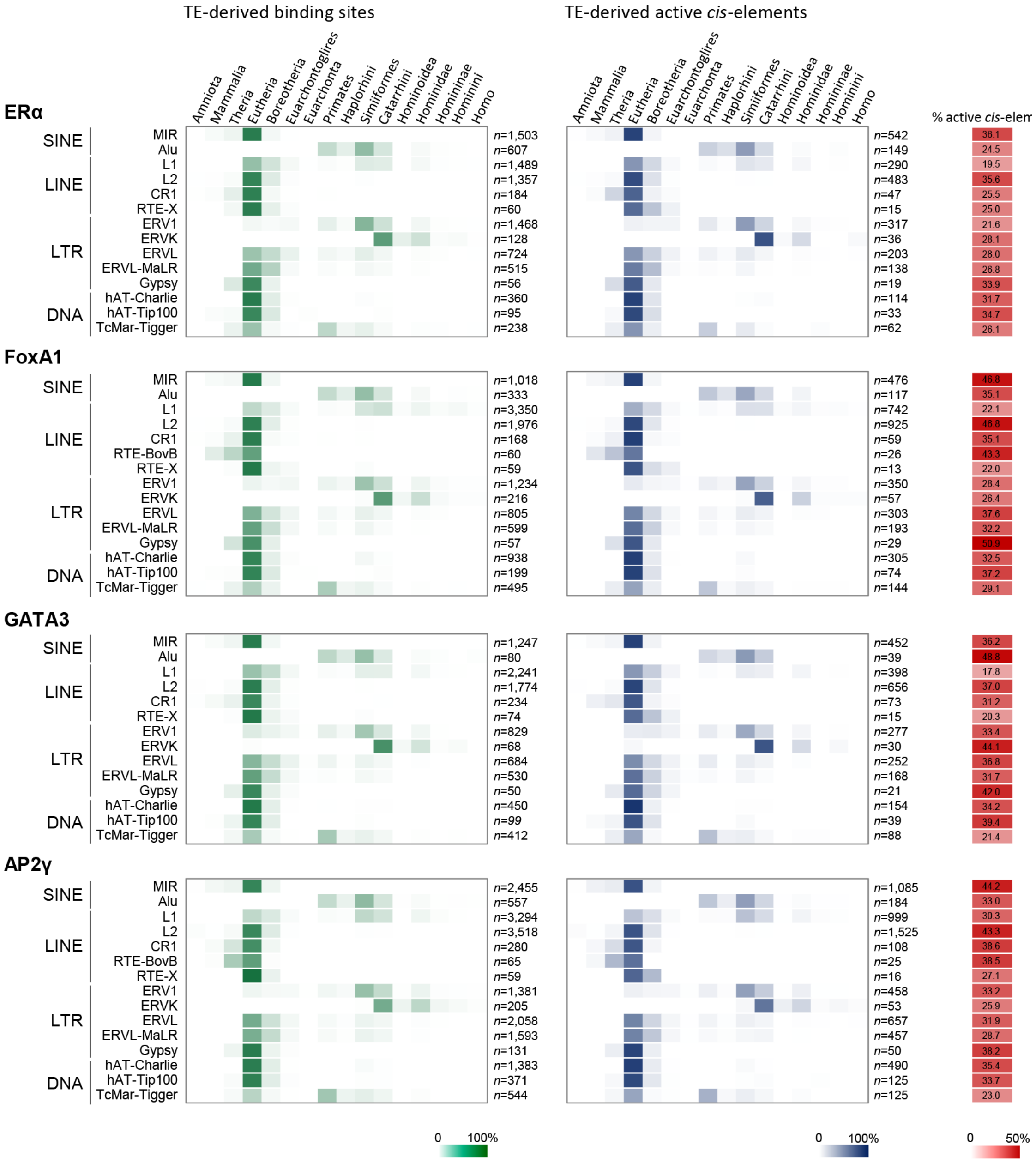

Figure S8. Estimated time of acquisition of the transcription factor binding sequences on TEs during mammalian evolution. The proportions of binding sites were represented separately for each TE category for all TE-derived binding sites (green) and those annotated as active *cis*-elements (active promoters or strong enhancers by ChromHMM) (blue). Proportion of the active *cis*-elements among all binding sites are shown in right (red).

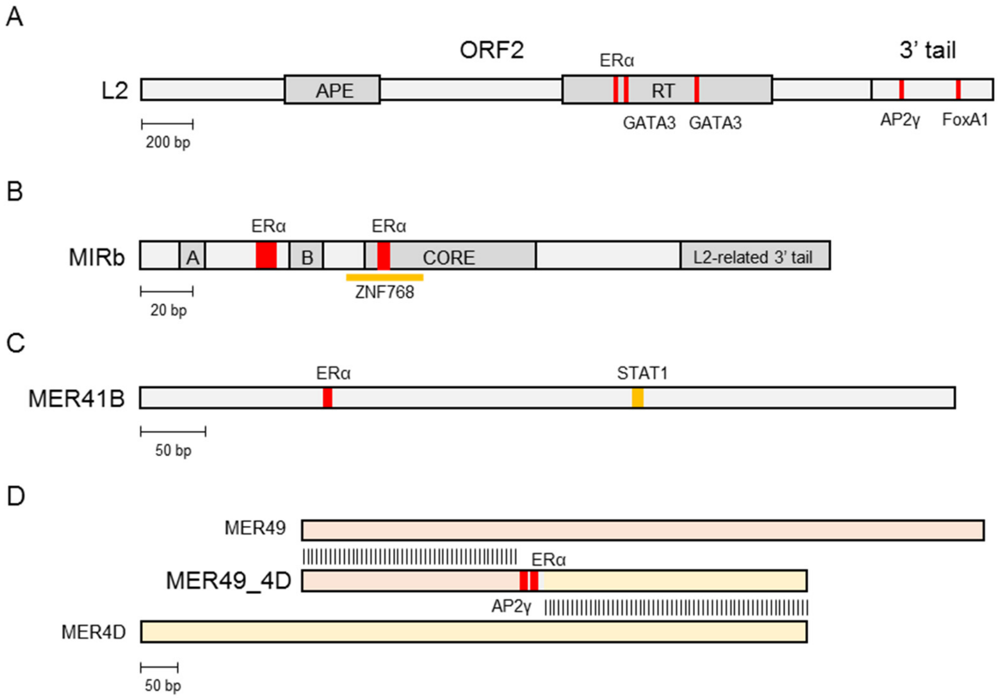

Figure S9. Schematic representation of representative ERα-binding TEs. (**A–D**) Positions of the transcription factor binding motifs were mapped on representative TEs shown in Figure 2A: (**A**) L2, (**B**) MIRb, (**C**) MER41B, and (**D**) MER49_4D. Red bars denote positions of transcription factor binding sites. Alignments of the binding sequences are shown in Supplementary Figure S10. APE, apurinic/apyrimidinic endonuclease domain; RT, reverse-transcriptase domain. In (**B**), A and B denote Box A and B of RNA pol III promoters, respectively, and CORE represents the central CORE domain shared among other SINEs in animals (4). Yellow bars in (**B**) and (**C**) represent the position of the ZNF768 and STAT1 binding motifs in MIR (5) and MER41B (6), respectively. In (**D**), note that MER49_4D is a hybrid LTR of MER49 and MER4D from their former and latter regions, respectively, and the ERα and AP2γ binding motifs are located within the intervening region.

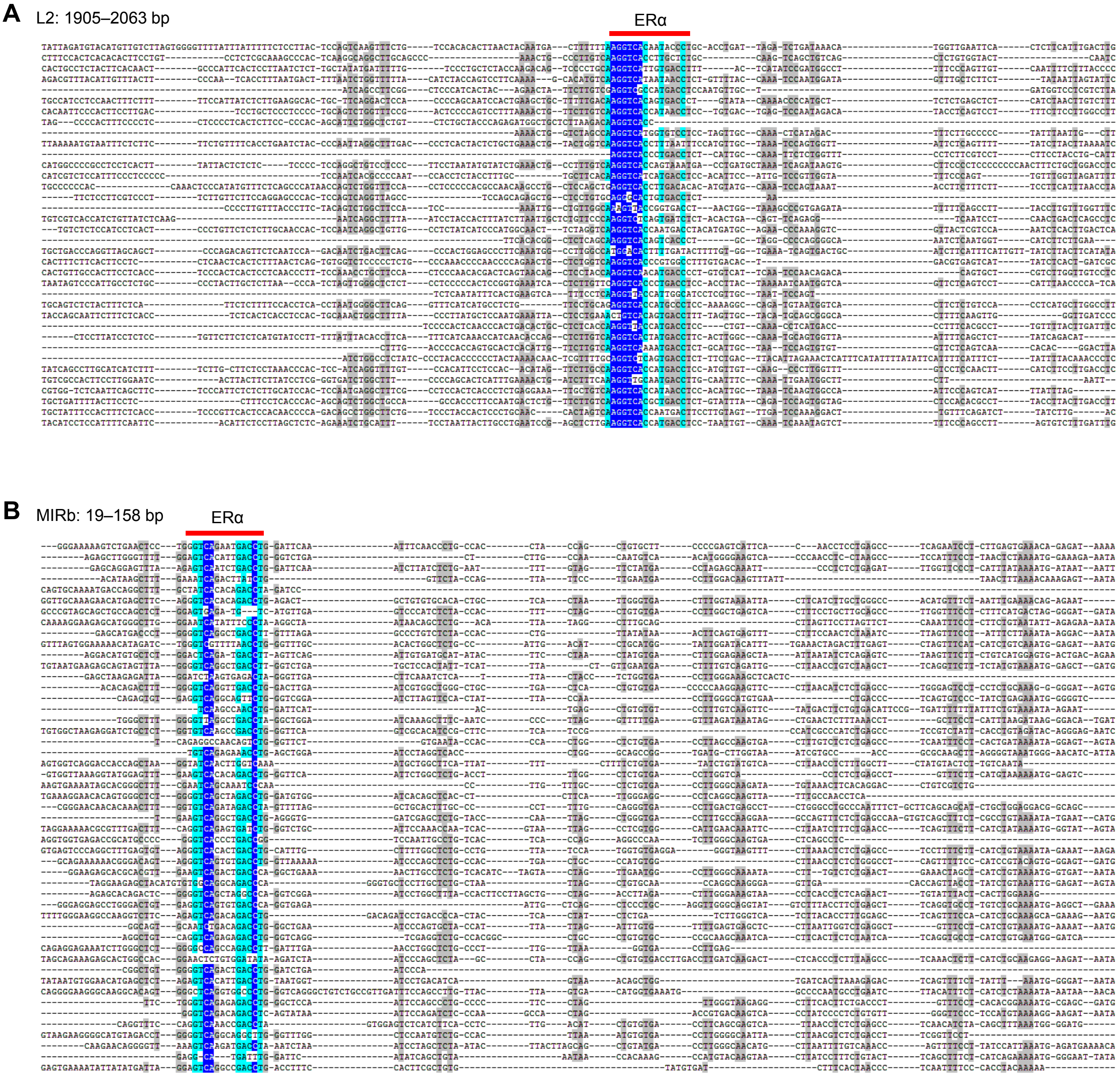
A
L2: 1905–2063 bp
ERα
B
MIRb: 19–158 bp
ERα

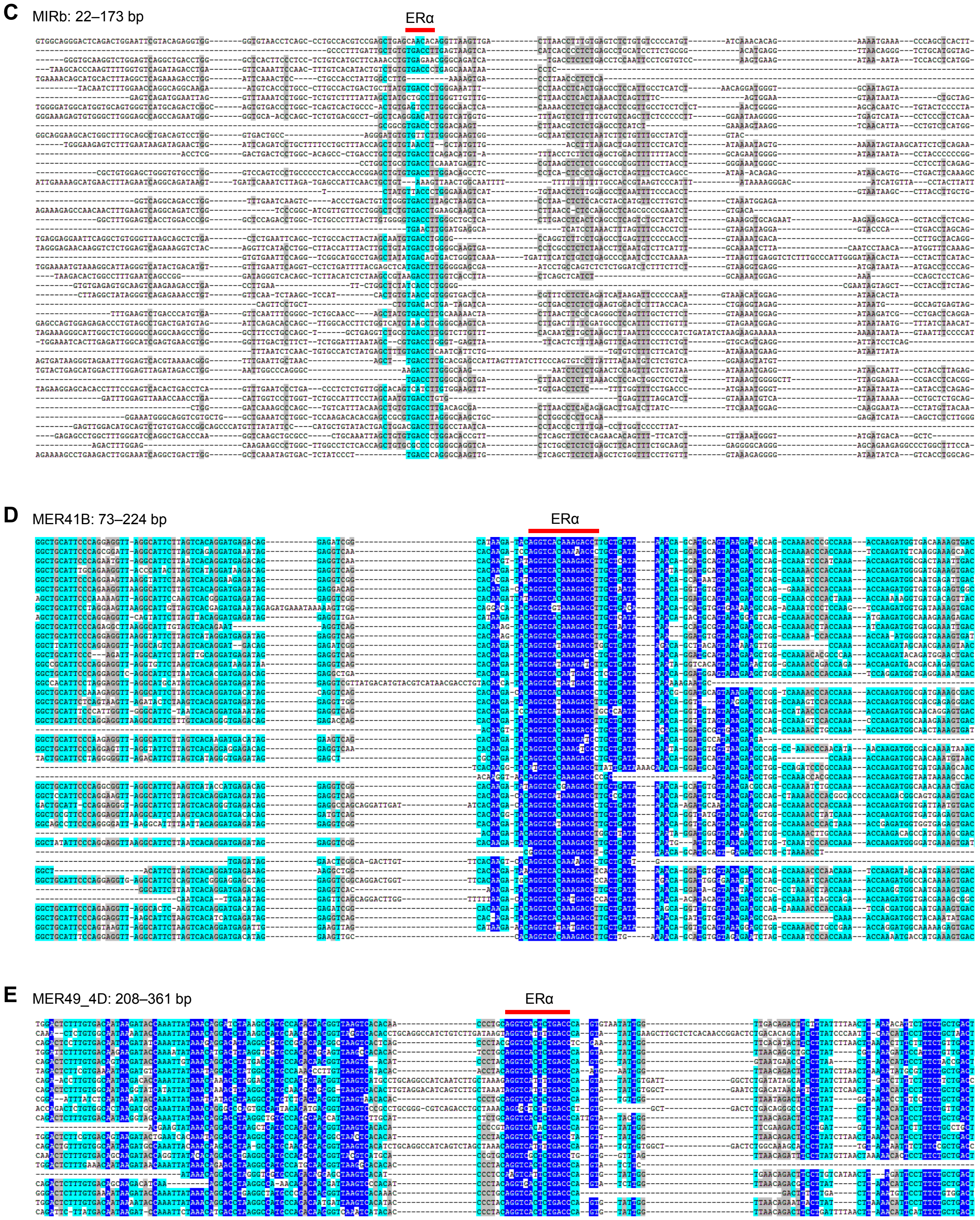
C
MIRb: 22–173 bp
ERα
D
MER41B: 73–224 bp
ERα
E
MER49_4D: 208–361 bp
ERα

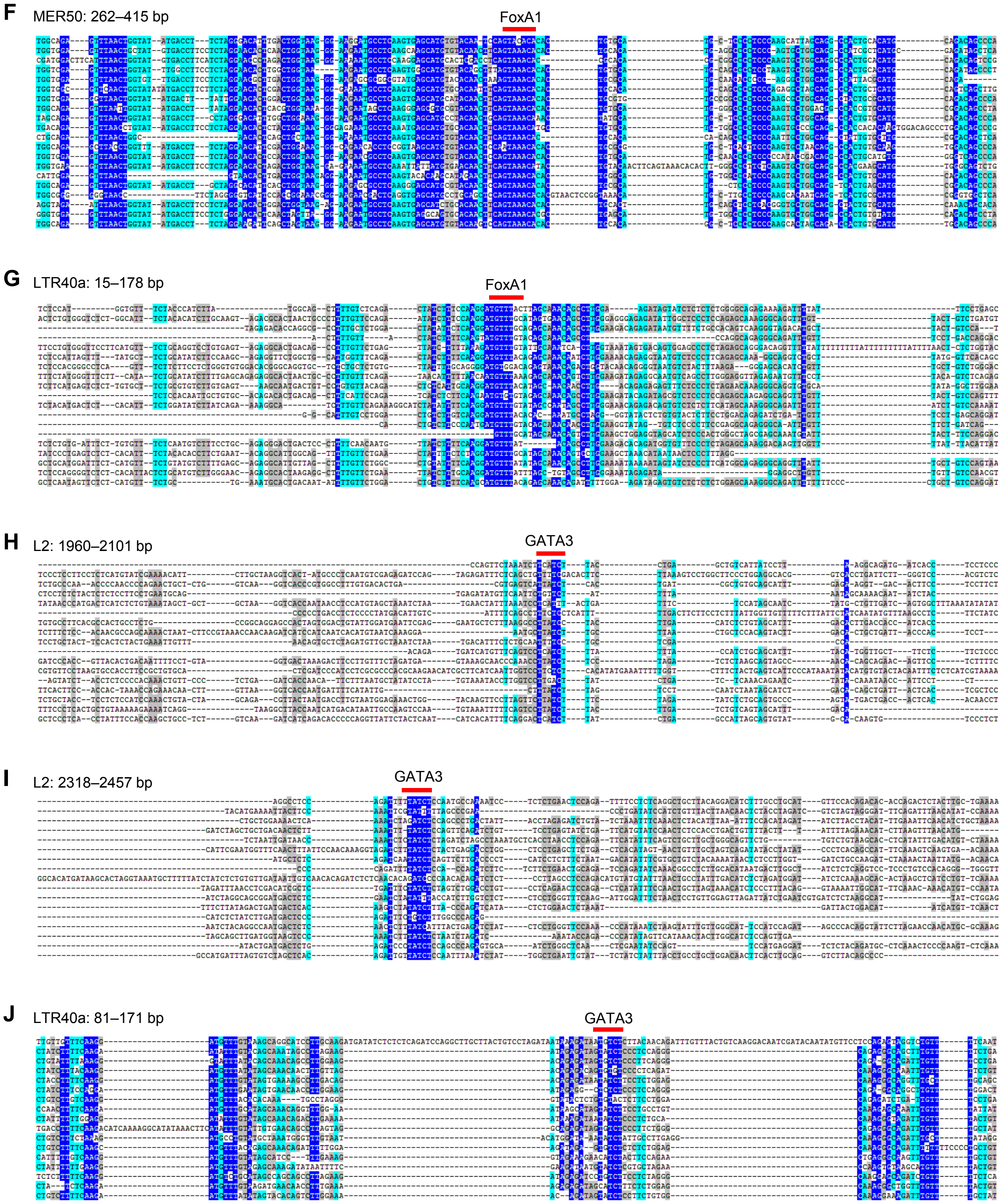
F
MER50: 262–415 bp
FoxA1
G
LTR40a: 15–178 bp
FoxA1
H
L2: 1960–2101 bp
GATA3
I
L2: 2318–2457 bp
GATA3
J
LTR40a: 81–171 bp
GATA3

K L2a_3end: 77–187 bp

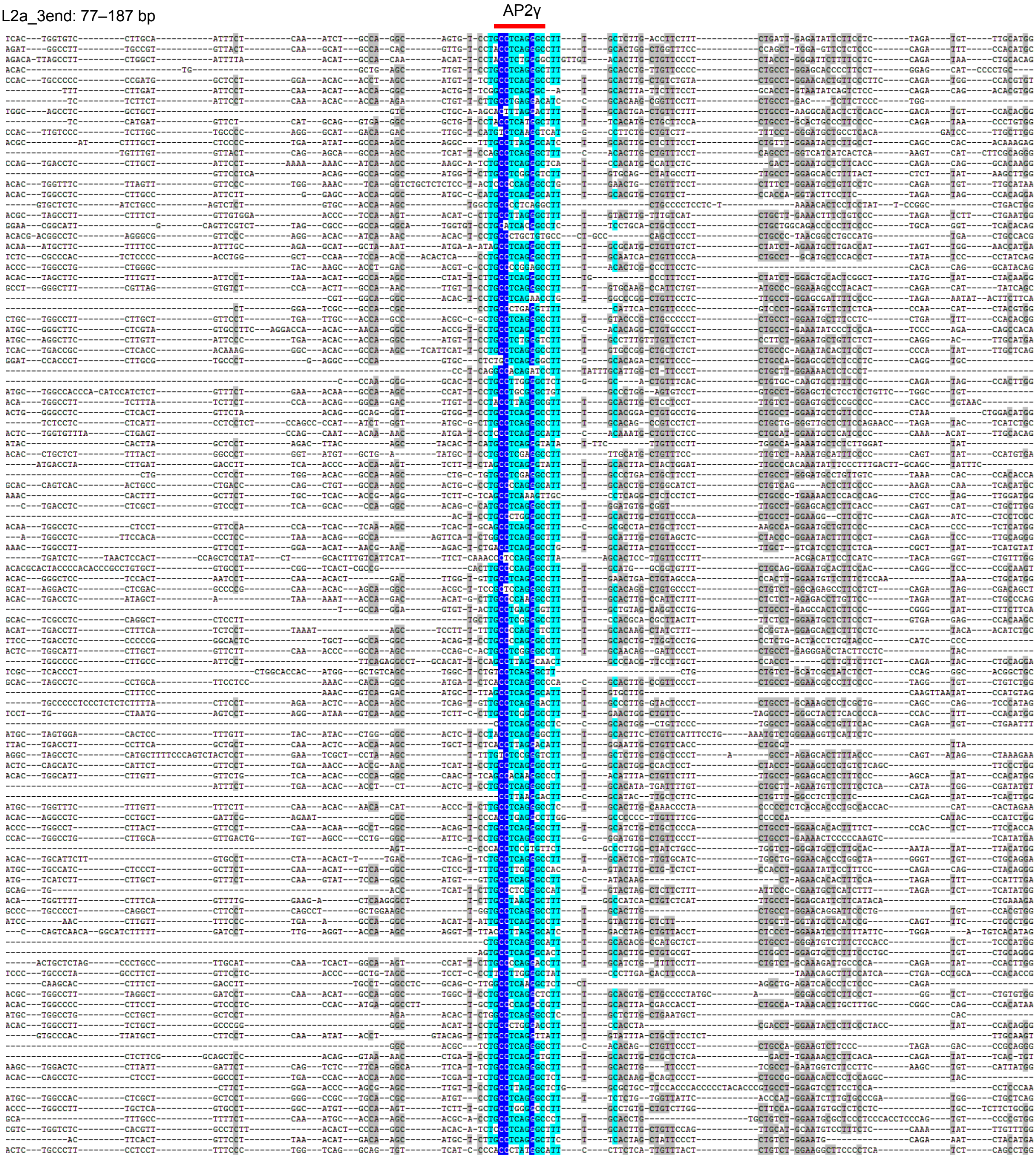

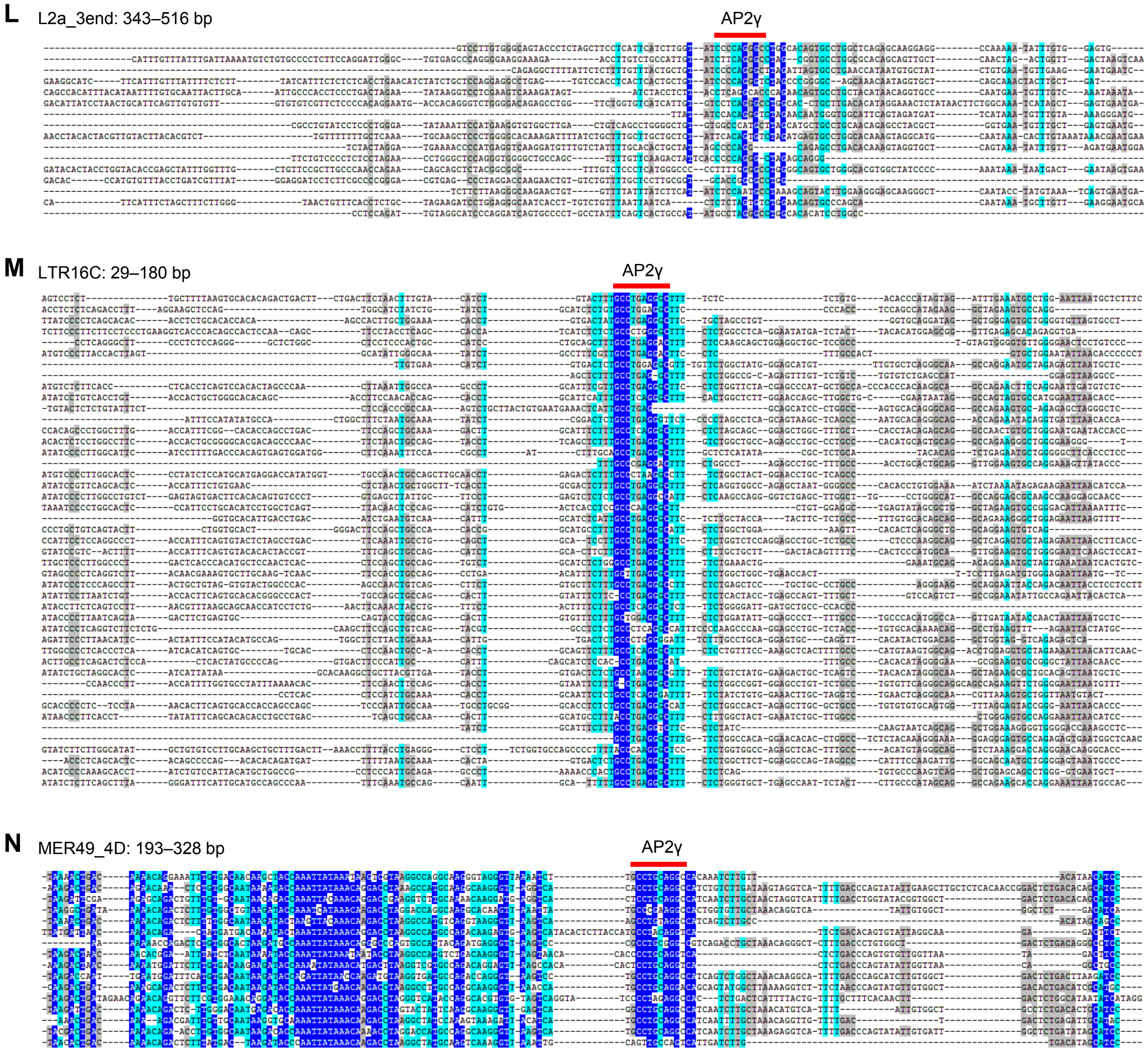

Figure S10. Alignments of TE sequences bound by transcription factors. (**A–E**) ERα, (**F–G**) FoxA1, (**H–J**) GATA3, and (**K–N**) AP2γ. Locations of these binding sites on the consensus sequence show the explicit peaks as in Fig. 2. Blue, light blue, and grey backgrounds represent >90%, >75%, and >60% shared nucleotides among the sequences, respectively. Conserved binding motifs are shown by red bars.

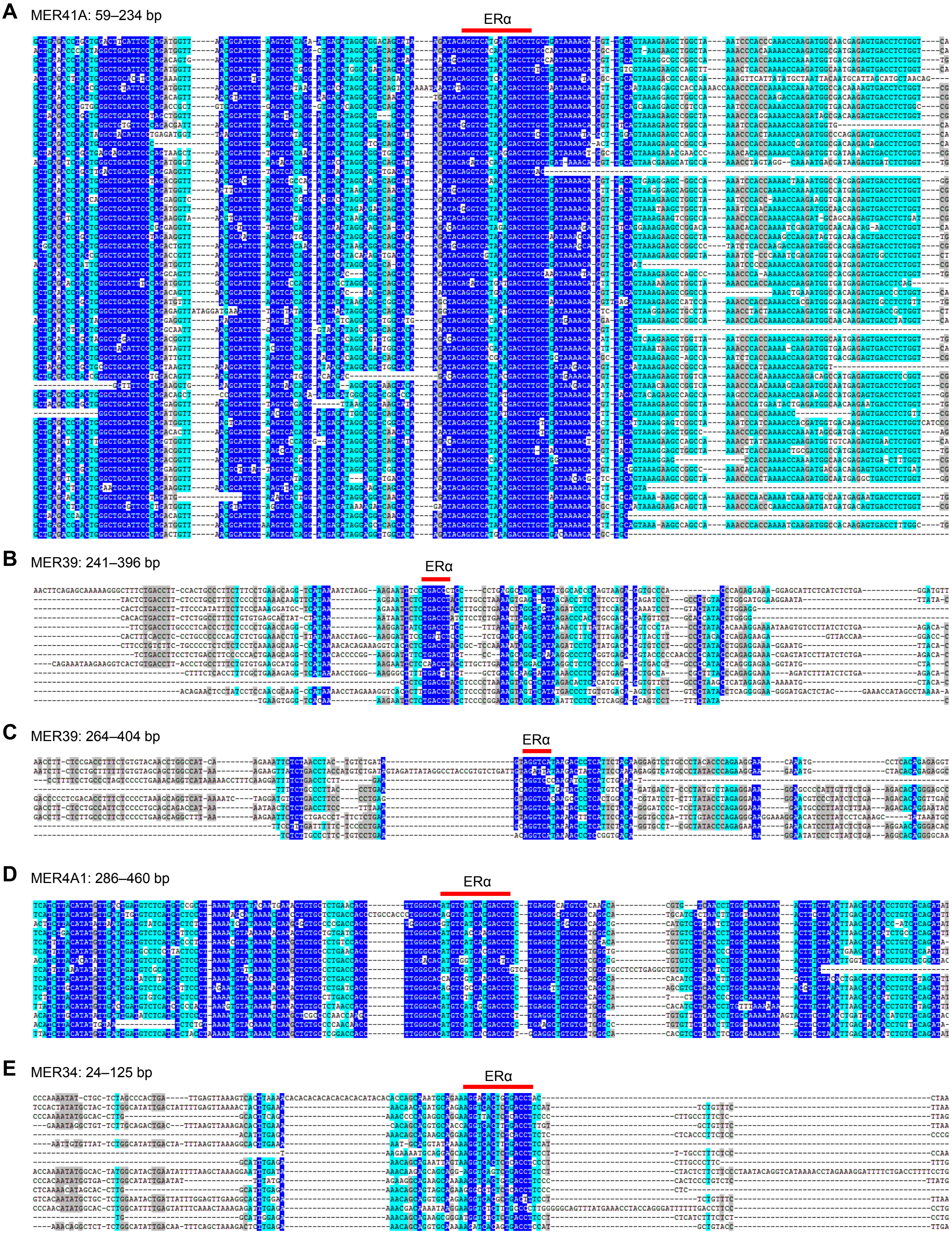
A
MER41A: 59–234 bp
ERα
B
MER39: 241–396 bp
ERα
C
MER39: 264–404 bp
ERα
D
MER4A1: 286–460 bp
ERα
E
MER34: 24–125 bp
ERα

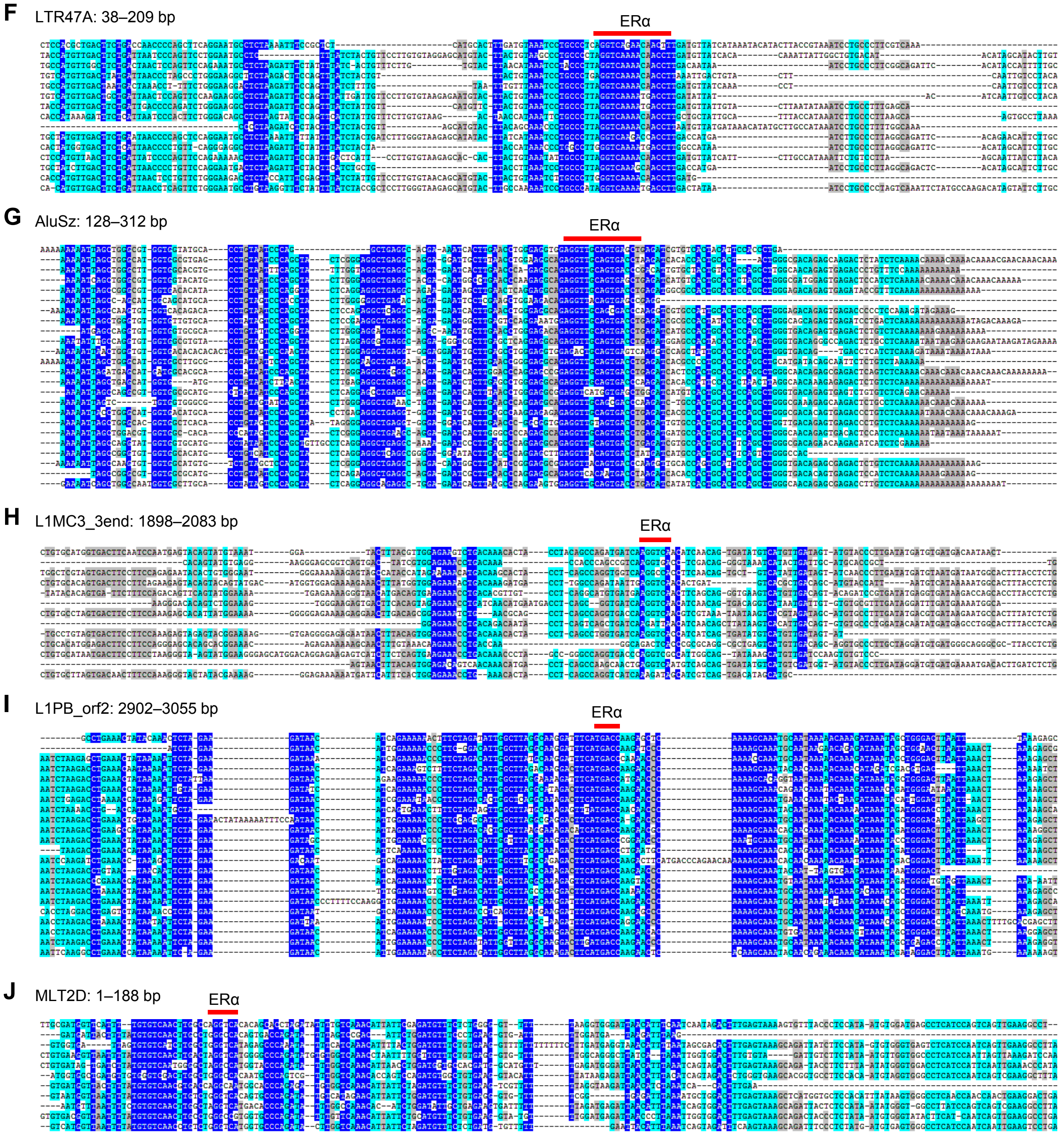

Figure S11. Alignments of TE sequences with ERα binding sites from Figures S4 and S5. Blue, light blue, and grey backgrounds represent >90%, >75%, and >60% shared nucleotides among the sequences, respectively. Conserved binding motifs are shown by red bars.

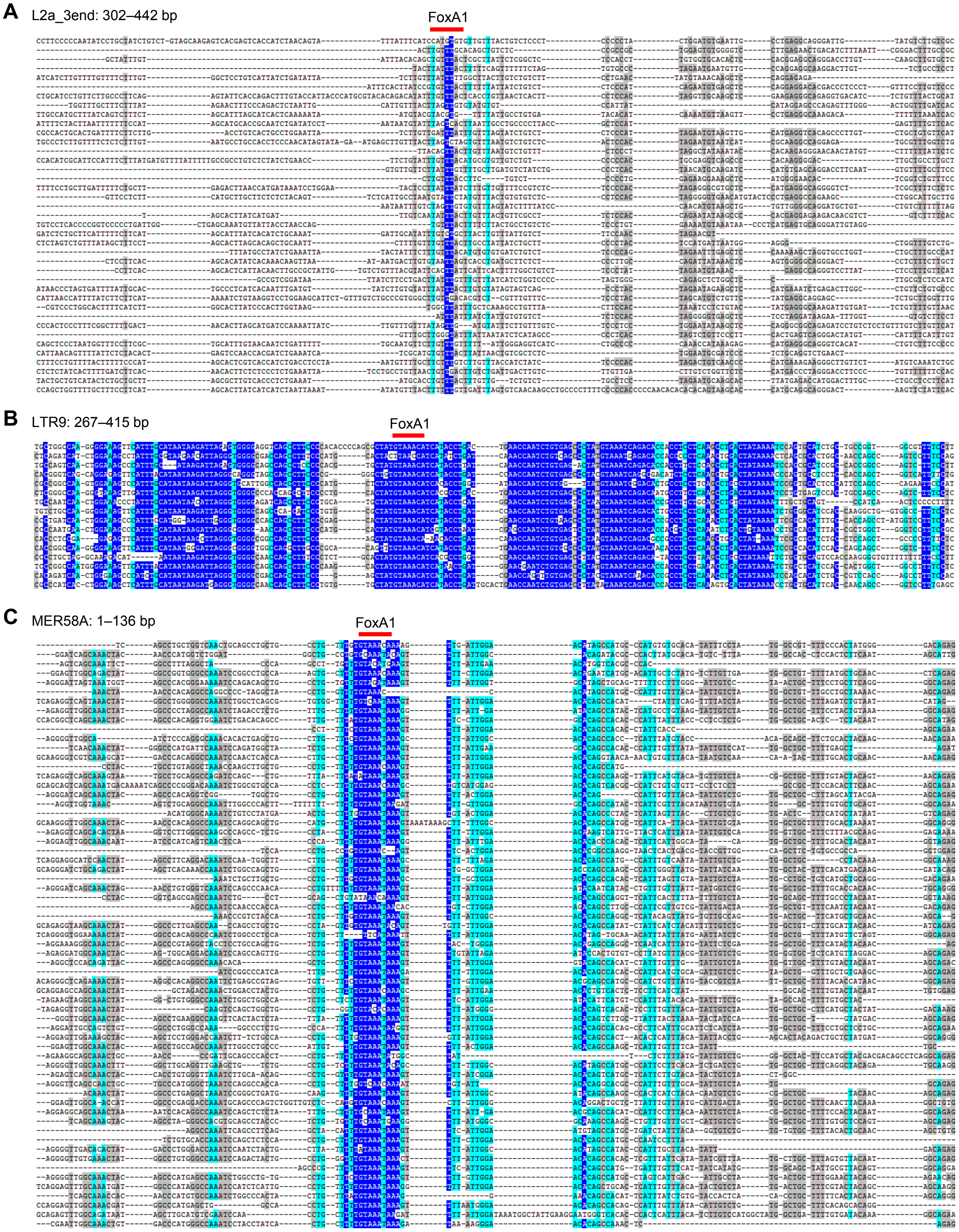
A
L2a_3end: 302–442 bp
FoxA1
B
LTR9: 267–415 bp
FoxA1
C
MER58A: 1–136 bp
FoxA1

D MER58A: 1–176 bp

FoxA1

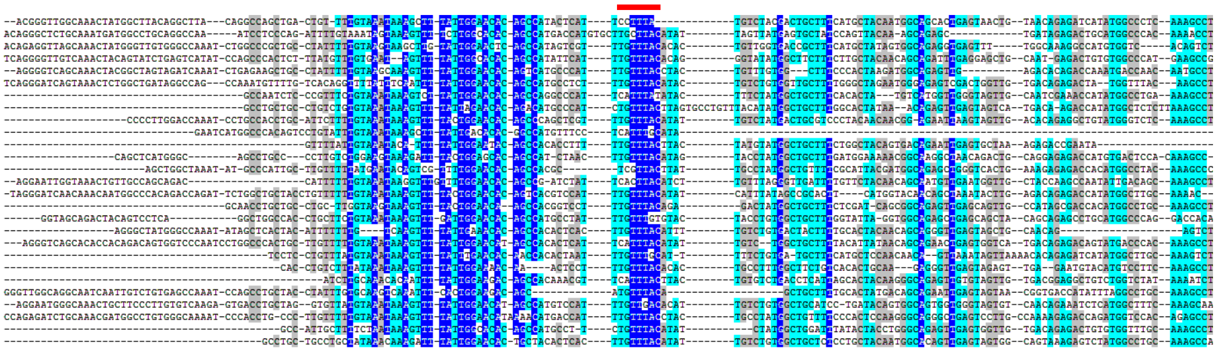

E MER1A: 31–191 bp

FoxA1

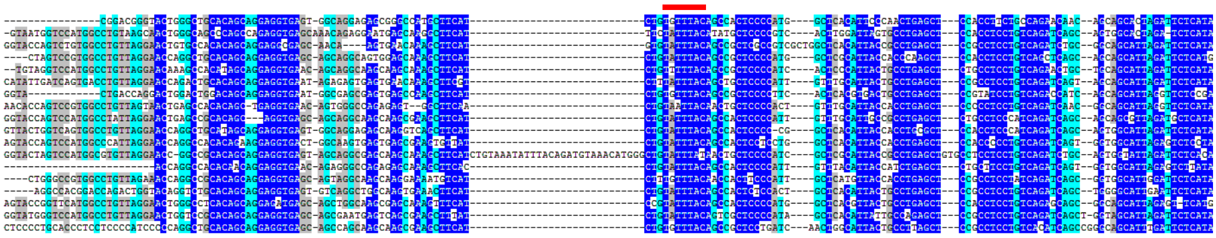

F Tigger3a: 164–348 bp

FoxA1

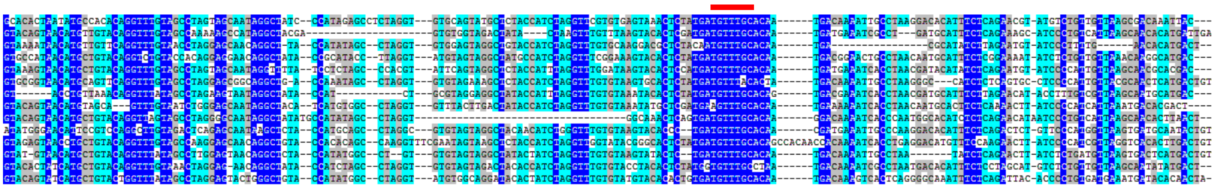

G Tigger3b: 1105–1231 bp

FoxA1

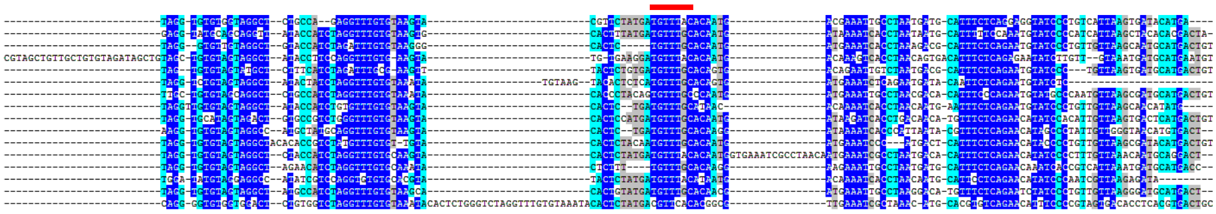

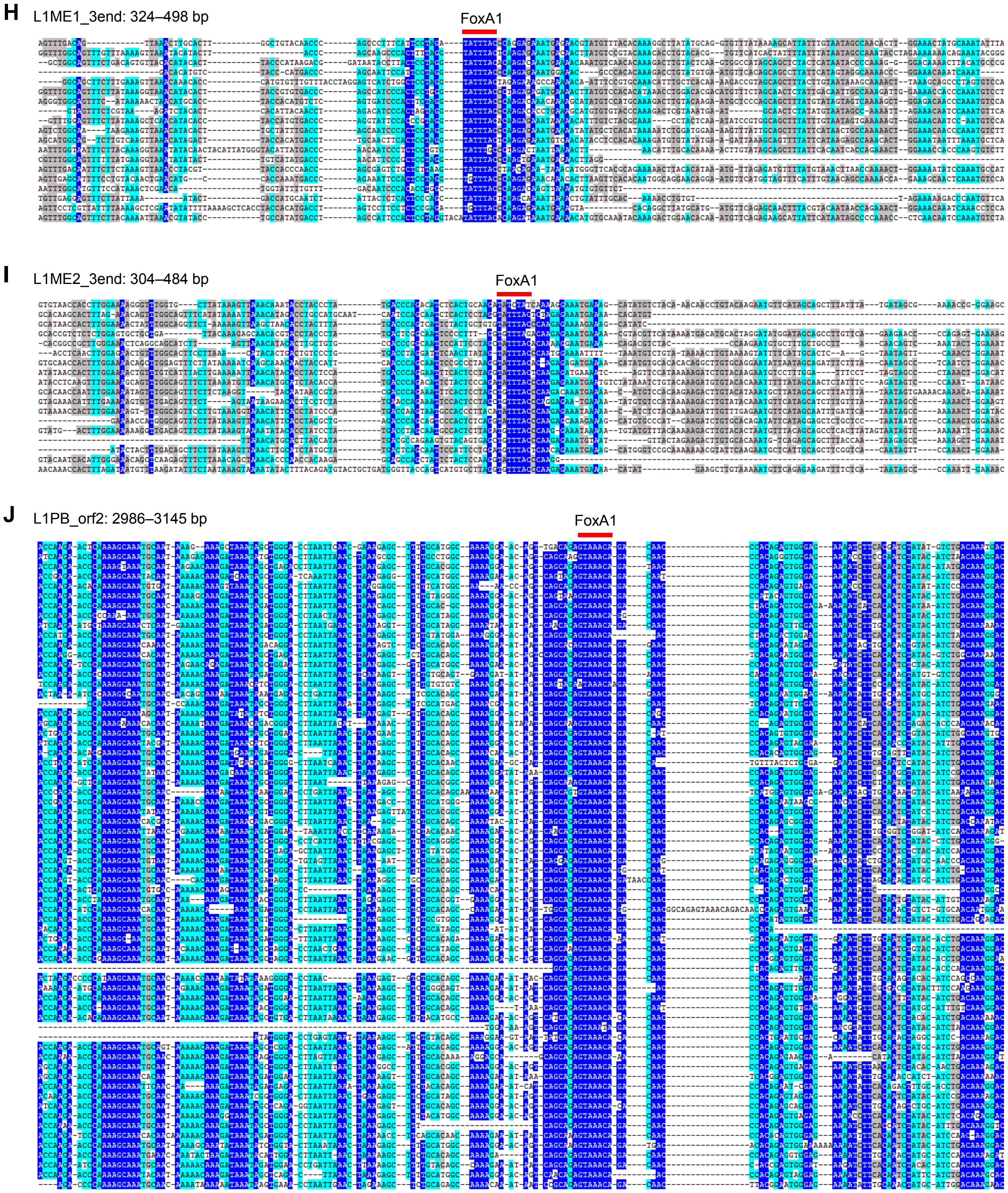
H
L1ME1_3end: 324–498 bp
FoxA1
I
L1ME2_3end: 304–484 bp
FoxA1
J
L1PB_orf2: 2986–3145 bp
FoxA1

K L1PREC2_orf2: 3029–3182 bp

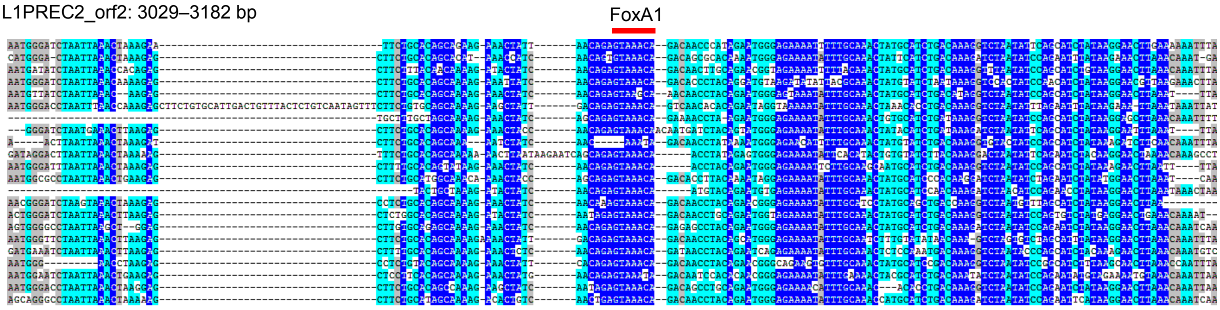

L HERVH: 5632–5794 bp

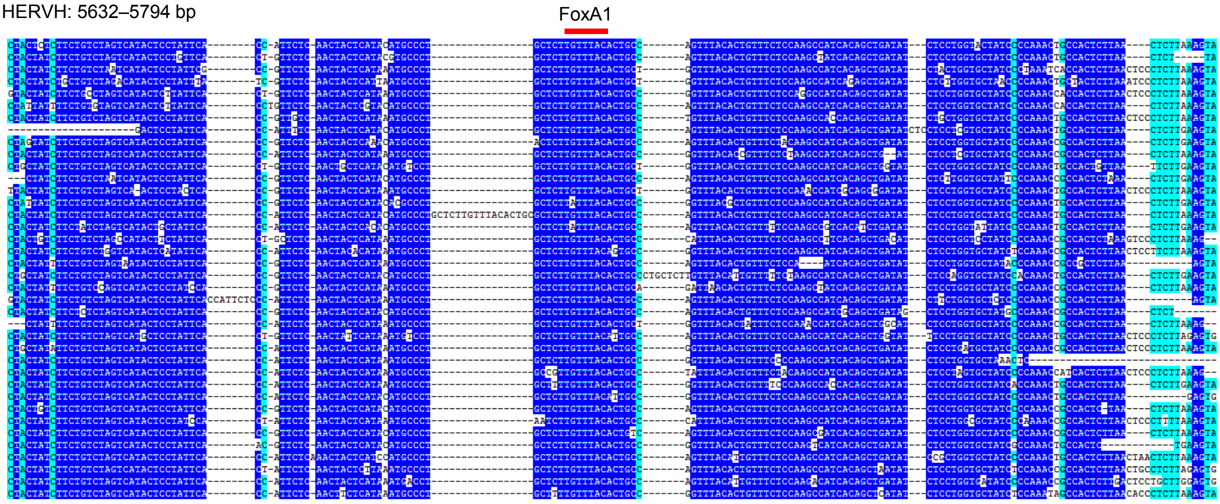

Figure S12. Alignments of the TE sequences with FoxA1 binding sites from Figures S4 and S5. Blue, light blue, and grey backgrounds represent >90%, >75%, and >60% shared nucleotides among the sequences, respectively. Conserved binding motifs are shown by red bars.

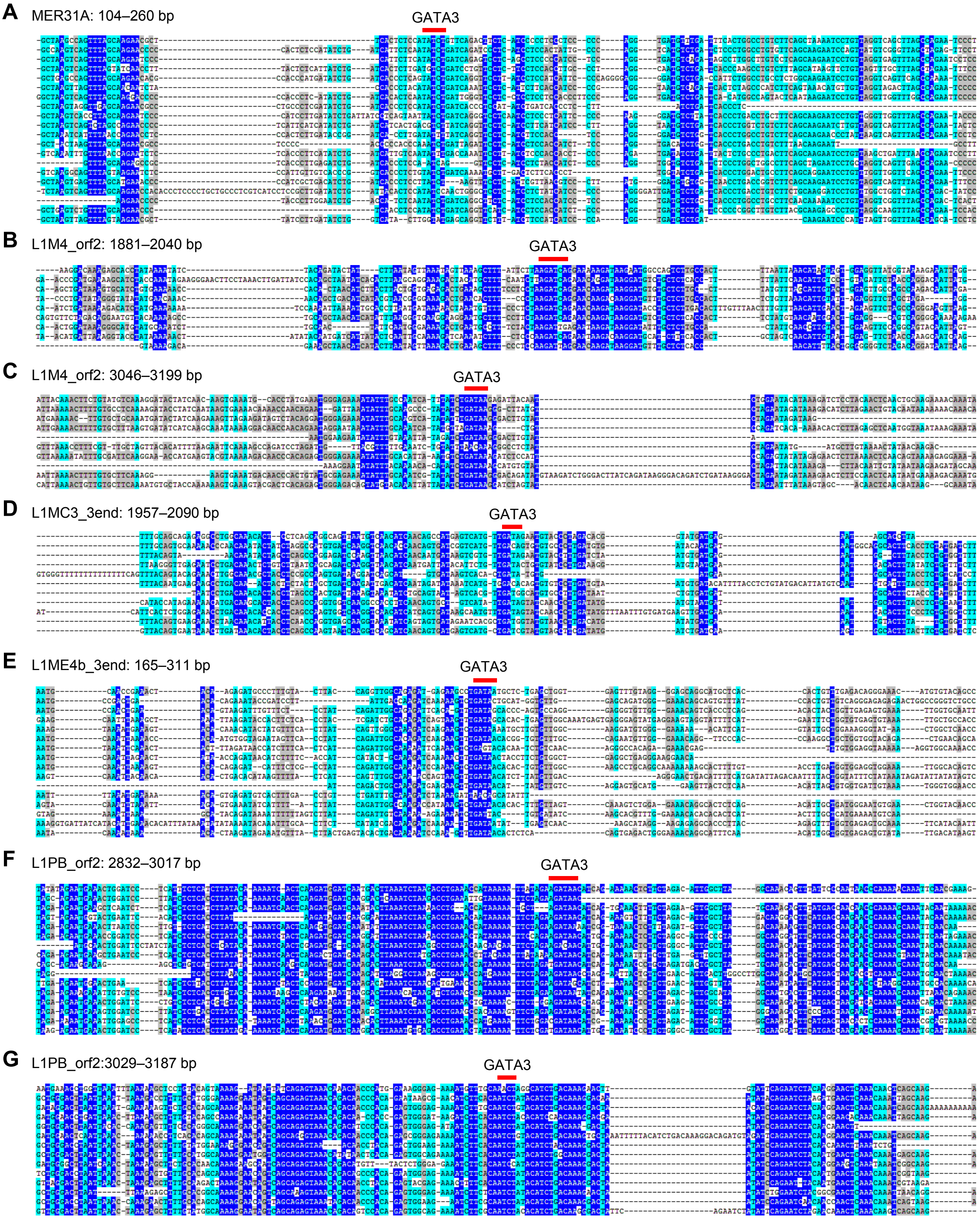

Figure S13. Alignments of TE sequences with GATA3 binding sites from Figures S4 and S5. Blue, light blue, and grey backgrounds represent >90%, >75%, and >60% shared nucleotides among the sequences, respectively. Conserved binding motifs are shown by red bars.

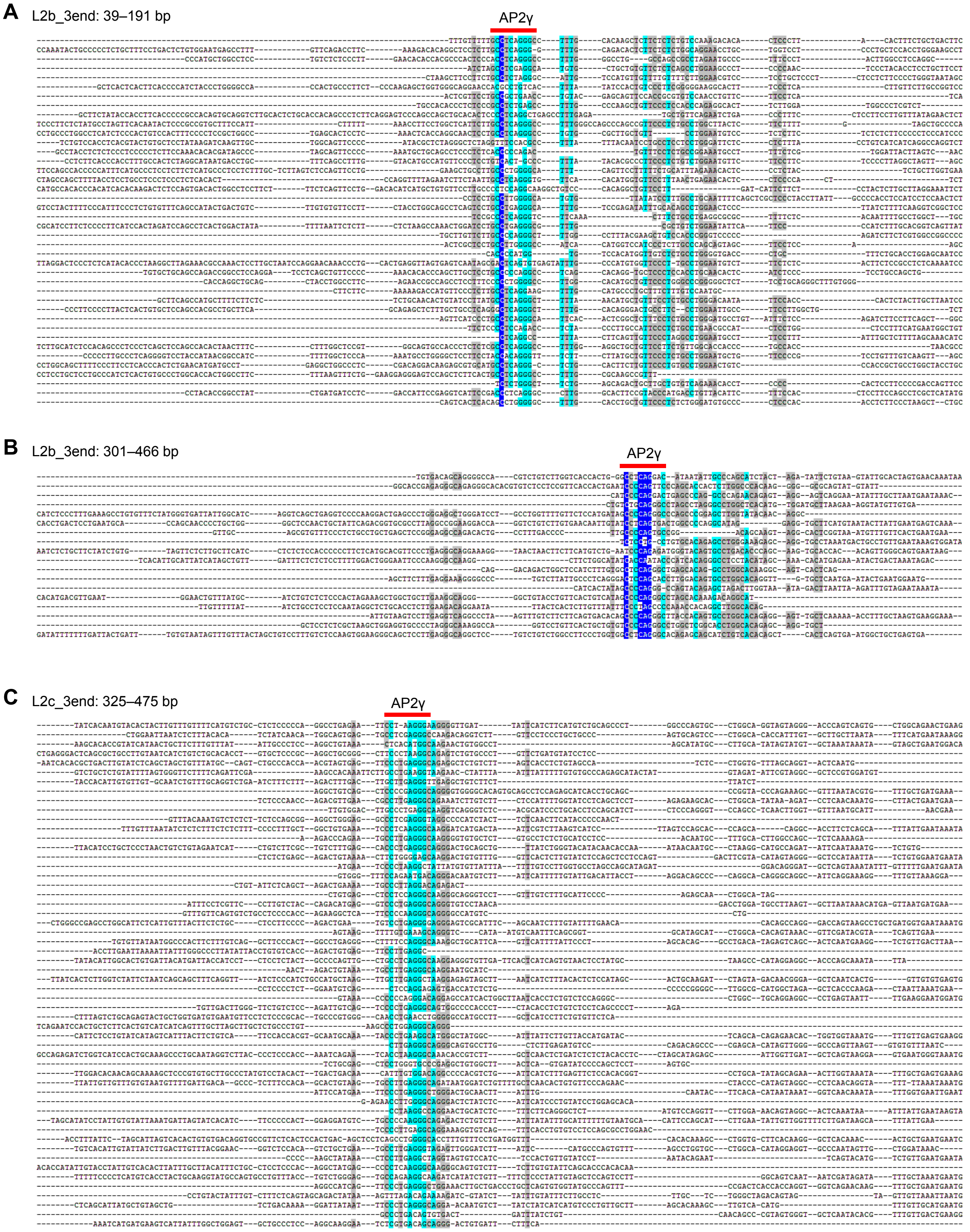
A
L2b_3end: 39–191 bp
AP2γ
B
L2b_3end: 301–466 bp
AP2γ
C
L2c_3end: 325–475 bp
AP2γ

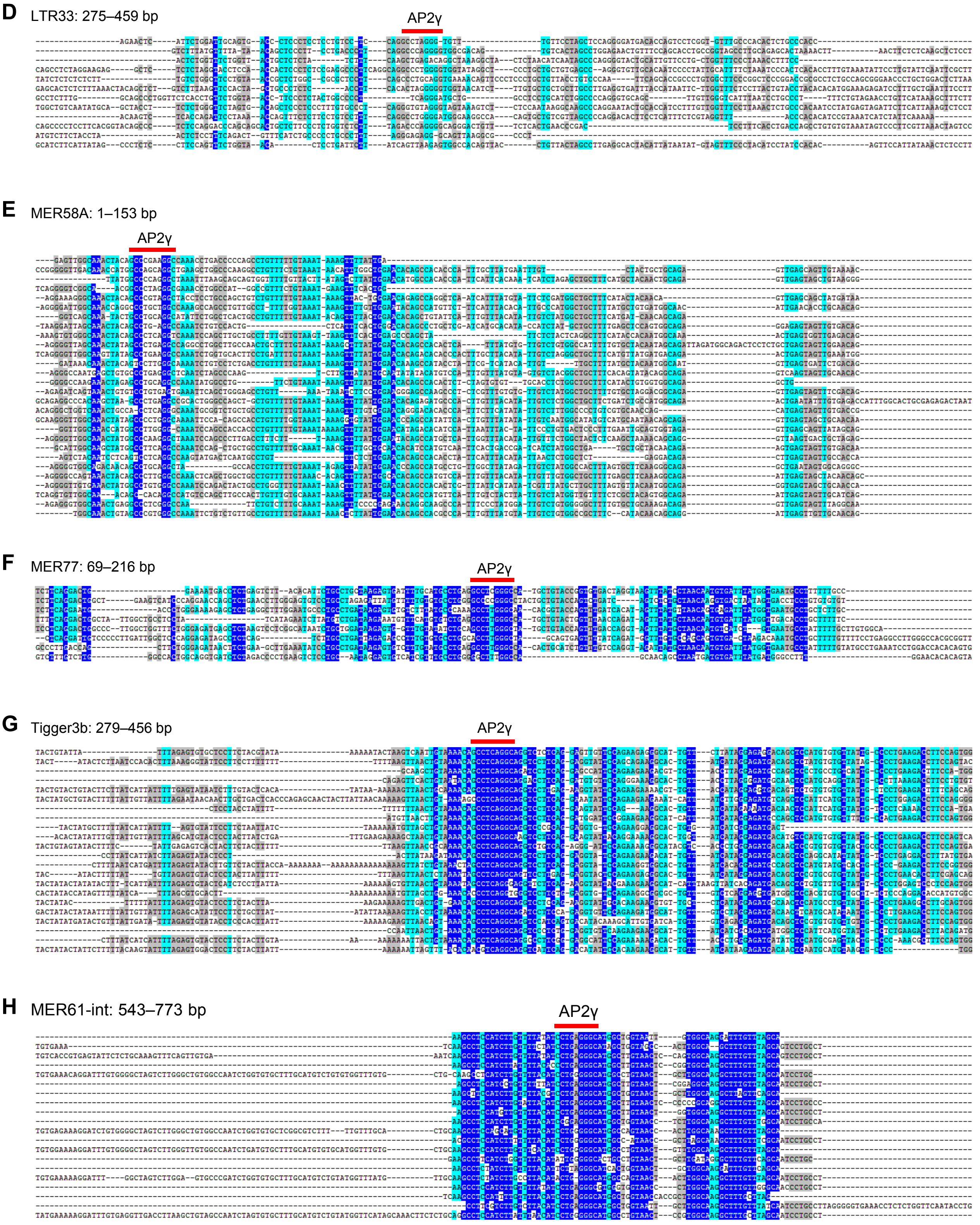
D
LTR33: 275–459 bp
AP2γ
E
MER58A: 1–153 bp
AP2γ
F
MER77: 69–216 bp
AP2γ
G
Tigger3b: 279–456 bp
AP2γ
H
MER61-int: 543–773 bp
AP2γ

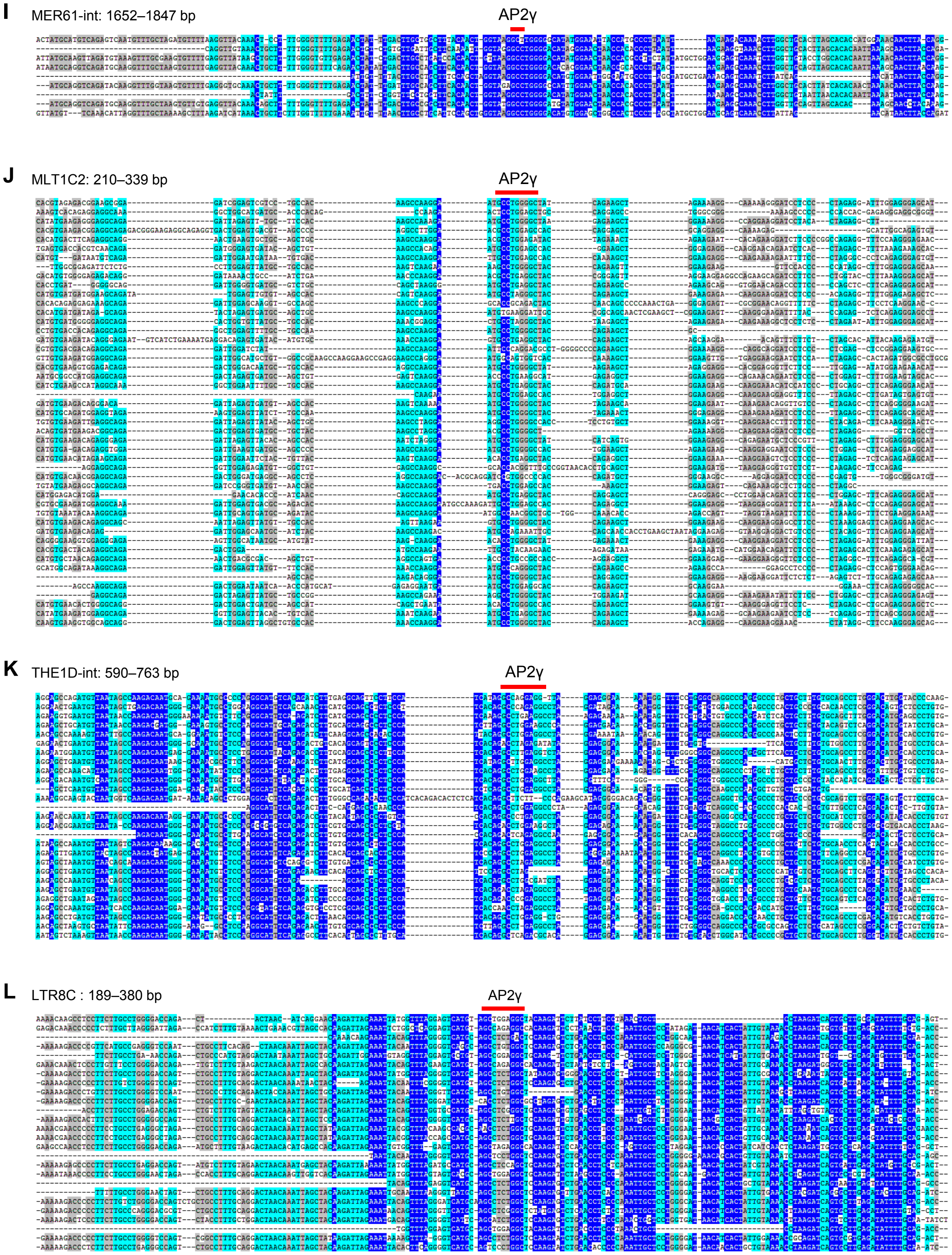
I
MER61-int: 1652–1847 bp
AP2γ
J
MLT1C2: 210–339 bp
AP2γ
K
THE1D-int: 590–763 bp
AP2γ
L
LTR8C : 189–380 bp
AP2γ

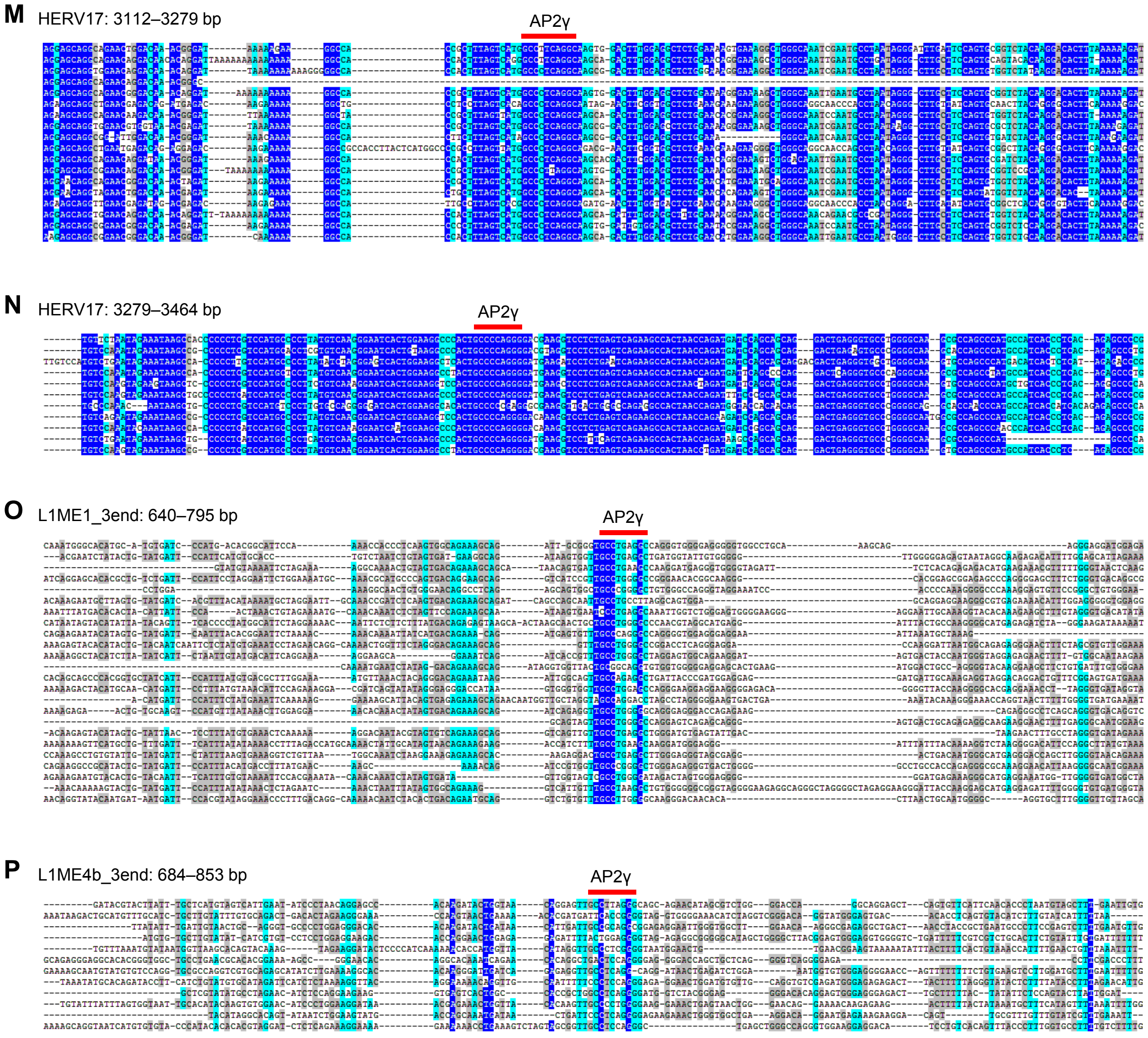

Figure S14. Alignments of TE sequences with AP2γ binding sites from Figures S4 and S5. Blue, light blue, and grey backgrounds represent >90%, >75%, and >60% shared nucleotides among the sequences, respectively. Conserved binding motifs are shown by red bars.

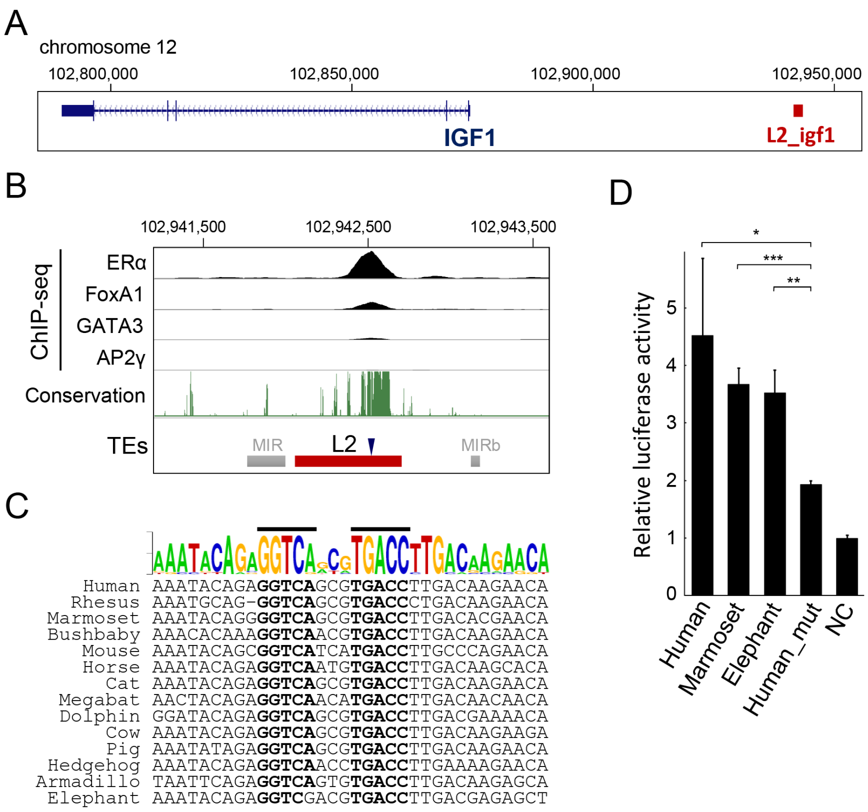

Figure S15. Enhancer function of an ERα-binding L2 element. (**A**) Location of the L2 locus (red) upstream of *Igf1* in human chromosome 12. (**B**) ChIP-seq peaks for ERα and the three pioneer factors (7–9) (min=0, max=150) as well as phastCons evolutionary conservation (from the UCSC Genome Browser; min=0, max=1.0). (**C**) Conservation of the ERα binding motif (bold) among eutherians in the L2 sequence denoted by an arrowhead in (**B**). (**D**) Relative luciferase activity of the human, marmoset, and elephant L2 sequences in MCF-7 cells. Human_mut represents the human sequence with a mutation in the ERα binding site. Data are shown as the mean ± SD (n = 3). Two-tailed t-test: *$p < 0.05$, **$p < 0.01$, ***$p < 0.001$. NC, negative control.

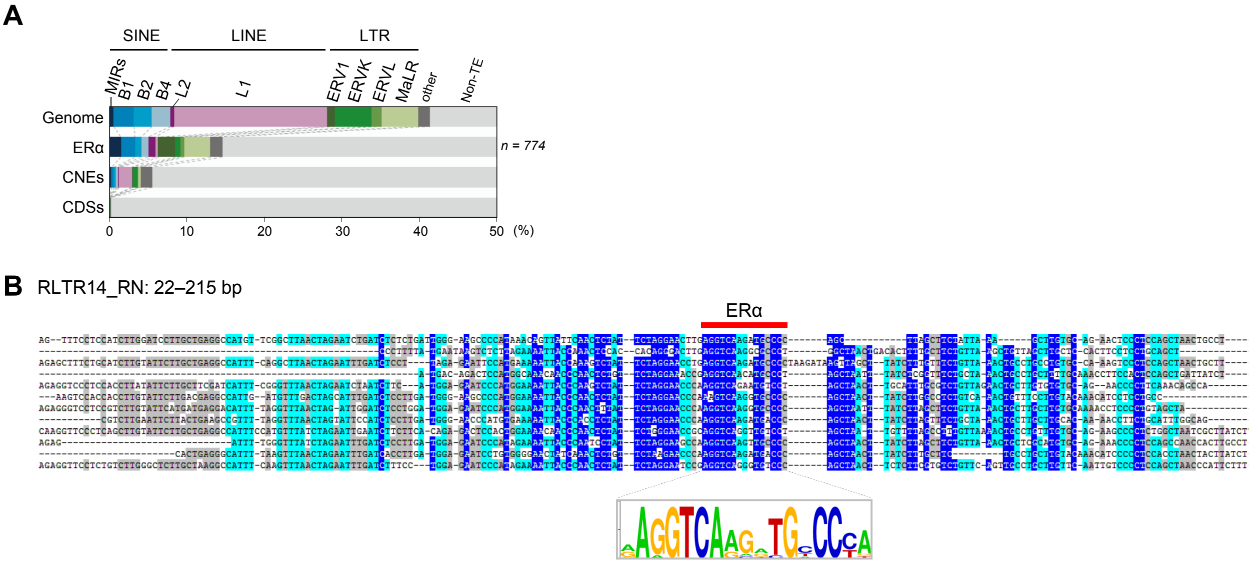

Figure S16. TEs bound by ERα in the mouse mammary gland tissue. (**A**) Percentage of TEs among the number of ERα-binding events. Proportions of TEs in the mouse genome (mm10, excluding chromosome Y) (Genome), conserved non-coding elements (CNEs), and protein-coding sequences (CDSs) are shown. SINE, short interspersed element; LINE, long interspersed element; LTR, long terminal repeat retrotransposon; DNA, DNA transposons. (**B**) Alignment of mouse RLTR14_RN sequences bound by ERα in the mammary gland tissue. Blue, light blue, and grey backgrounds represent >90%, >75%, and >60% shared nucleotides among the sequences, respectively. The conserved ERα-binding motif (logo) is shown below.

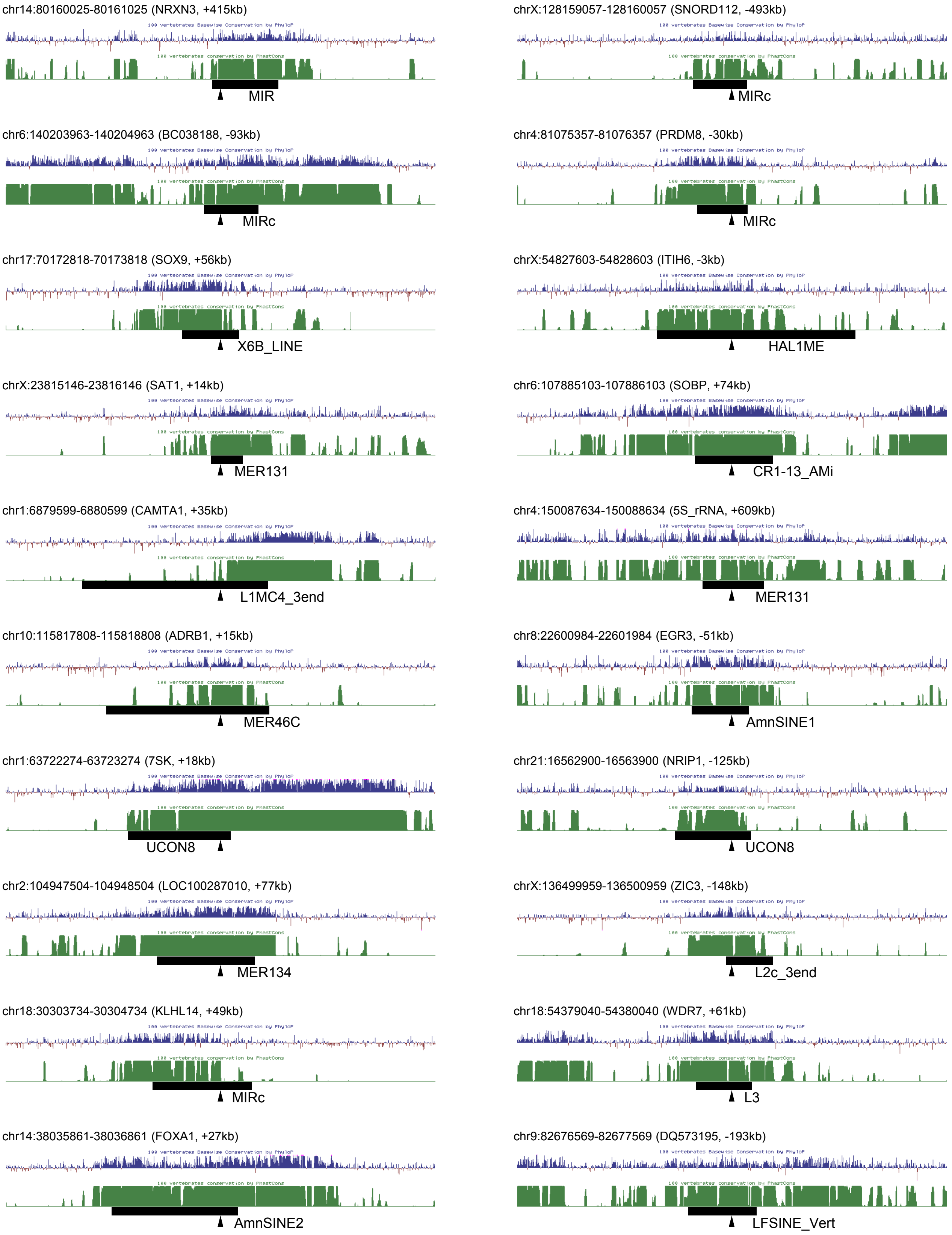

Figure S17. Representative TE-derived conserved regions bound by ERα. Bars and arrowheads indicate TEs and the ERα-binding sites, respectively. PhyloP (blue) and phastCons (green) conservation data were retrieved from the UCSC Genome Browser. The nearest gene and the distance are shown in parenthesis.

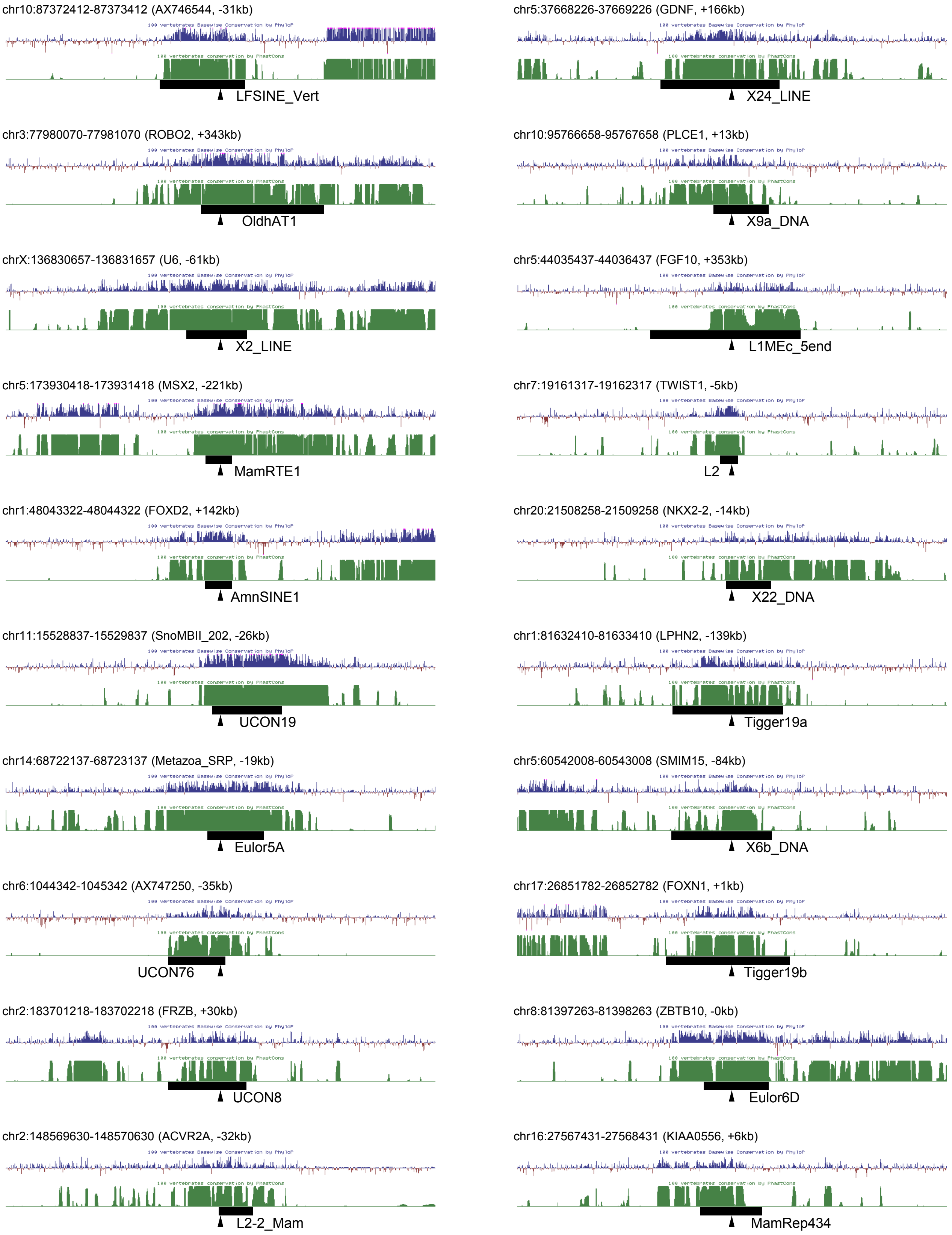

Figure S18. Representative TE-derived conserved regions bound by FoxA1. Bars and arrowheads indicate TEs and the FoxA1-binding sites, respectively. PhyloP (blue) and phastCons (green) conservation data were retrieved from the UCSC Genome Browser. The nearest gene and the distance are shown in parenthesis.

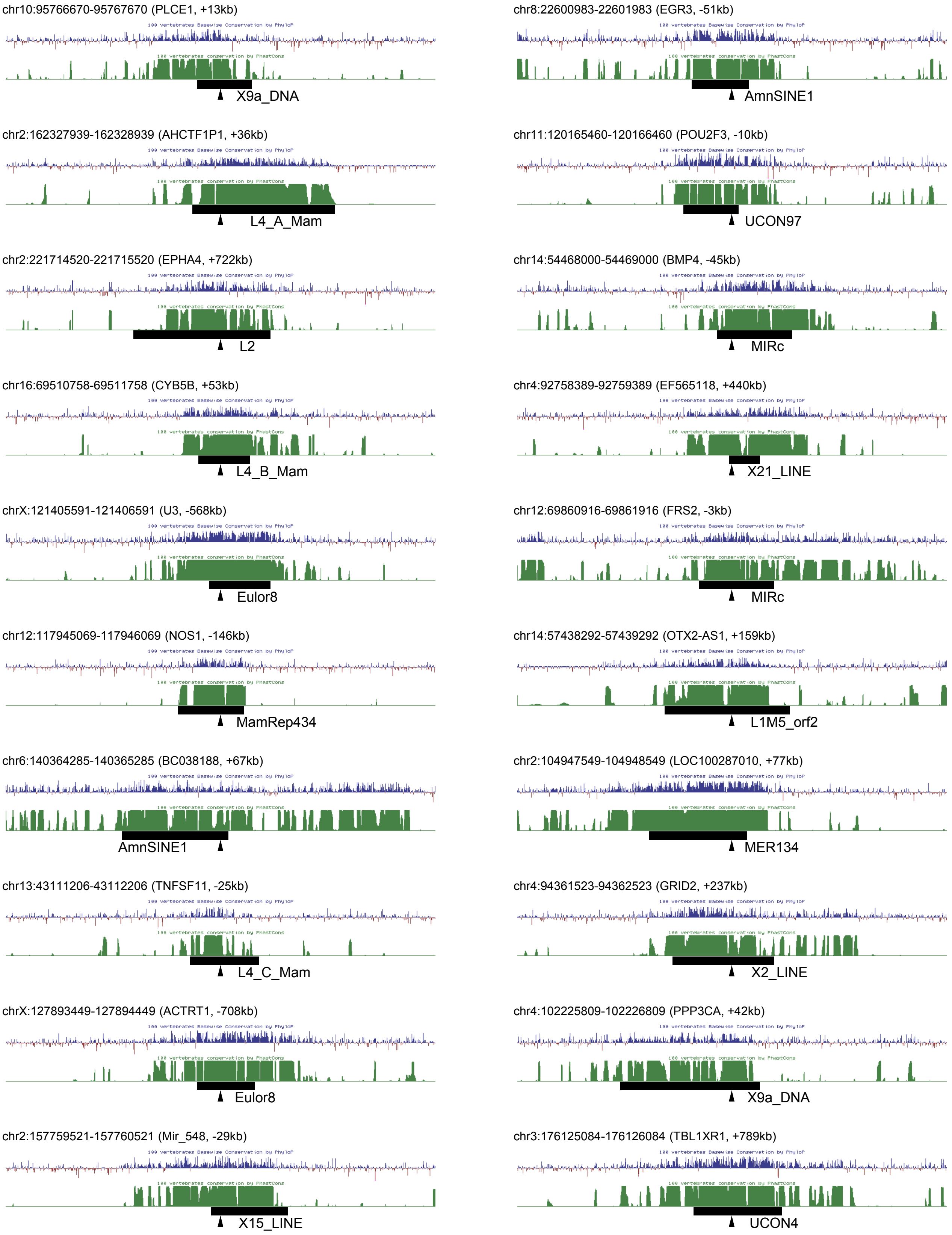

Figure S19. Representative TE-derived conserved regions bound by GATA3. Bars and arrowheads indicate TEs and the GATA3-binding sites, respectively. PhyloP (blue) and phastCons (green) conservation data were retrieved from the UCSC Genome Browser. The nearest gene and the distance are shown in parenthesis.

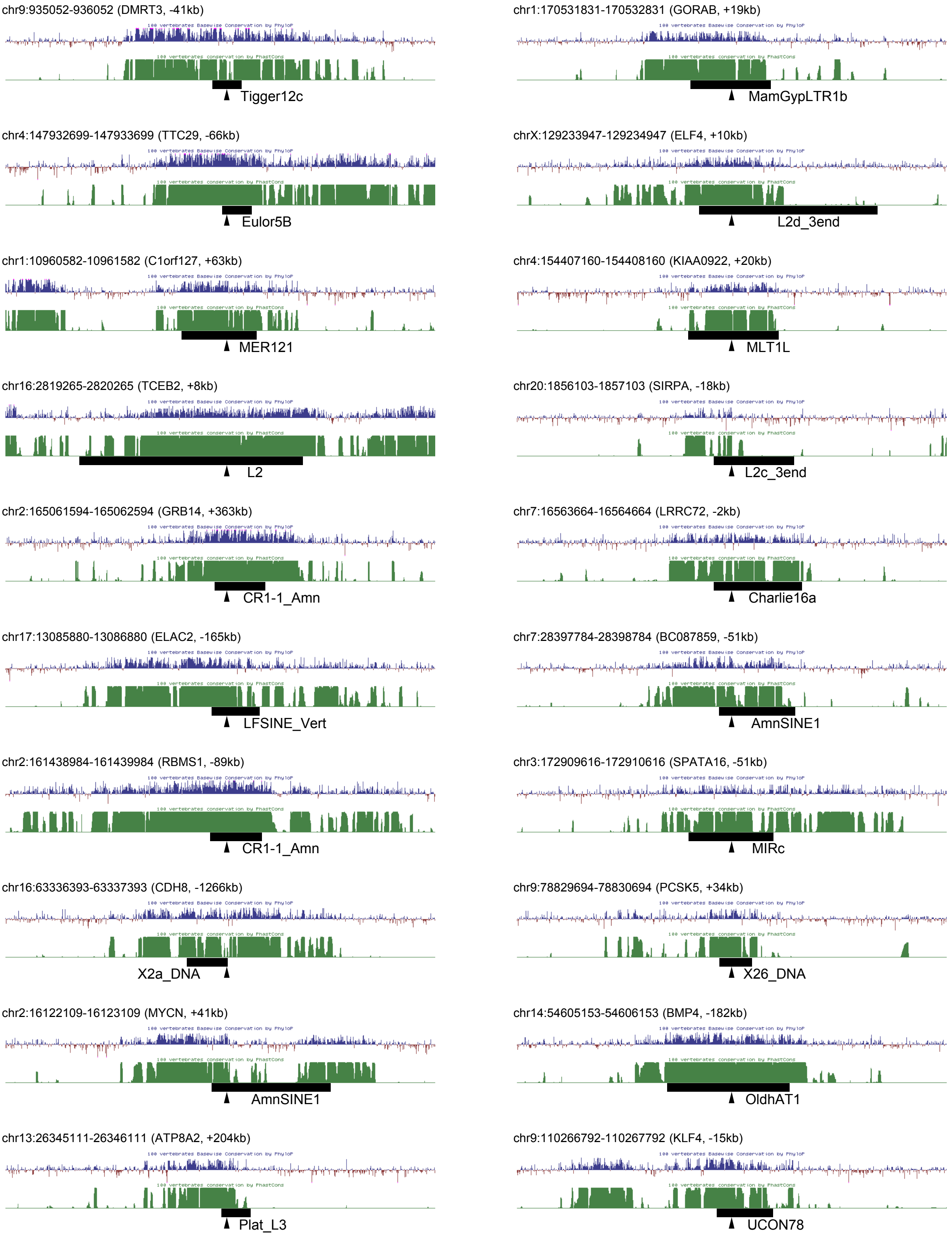

Figure S20. Representative TE-derived conserved regions bound by AP2γ. Bars and arrowheads indicate TEs and the AP2γ-binding sites, respectively. PhyloP (blue) and phastCons (green) conservation data were retrieved from the UCSC Genome Browser. The nearest gene and the distance are shown in parenthesis.

Table S1. Ten genes with known functions related to mammary gland development or breast cancer located within the region of highest TE density in human chromosome 20.

Gene	Location in human (hg19)	Function related to mammary gland or breast cancer
ZMYND8	chr20:45837858-45985633	ZMYND8 forms a complex with ERα and is involved in estrogen-induced transcriptional activation (10).
NCOA3	chr20:46130600-46285621	*Ncoa3*, also known as *Src-3*, is required for female reproductive function and normal mammary gland development, as revealed from the knockout mouse analysis (11). Human *Ncoa3* is associated with breast cancer risk (12).
SULF2	chr20:46286149-46414808	Upregulated expression in mammary gland tumours (13).
PREX1	chr20:47240792-47444420	High-frequency mutations are found in *PREX1* from human breast cancers (14).
STAU1	chr20:47729875-47804907	Nuclear interactome analysis suggests a possible preeminent role for STAU1 in the ERα network (15).
ZNFX1	chr20:47862438-47894756	*Zfas1*, an antisense long non-coding RNA to *Znfx1*, plays an important role in alveolar development and epithelial cell differentiation in the mammary gland (16).
SNAI1	chr20:48599512-48605420	*Snai1*, also known as *Snail1*, is involved in branching morphogenesis of mammary epithelial tissues (17).
CEBPB	chr20:48807119-48809227	*Cebpb* is essential for ductal morphogenesis and epithelial cell proliferation during mammary gland development (18–20).
PTPN1	chr20:49126857-49201300	*Ptpn1* is involved in the regulation of mammary alveologenesis and the expression of milk proteins (21), as well as mammary tumourigenesis (22).
BCAS4	chr20:49411430-49493714	*BCAS4* is overexpressed in breast cancers (23) and forms a fusion transcript with *BCAS3* (24).

Table S2. NCBI SRA accession numbers used in this study.

Transcription factor	ChIP	Control (input)	Ref.
ERα	SRR1635445, SRR1635446	SRR1635437, SRR1635438	7
FoxA1	SRR1635461, SRR1635462	SRR1635437, SRR1635438	7
GATA3	SRR540196, SRR540198, SRR540200, SRR540202, SRR540204	SRR540220	8
AP2γ	SRR039385	SRR039386	9
p300	SRR577809, SRR577810	SRR577916, SRR577917, SRR577918, SRR577919	25
H3K4me1	SRR1275469, SRR1275470	SRR1275475, SRR1275476	26
H3K4me3	SRR1275471, SRR1275472	SRR1275475, SRR1275476	26
H3K27ac	SRR1275473, SRR1275474	SRR1275475, SRR1275476	26
CTCF	SRR357511, SRR357512	SRR357466	27
ERα (mouse)	SRR647493	SRR647494	28

Supplementary References

1. Ernst, J., and Kellis, M. (2012) ChromHMM: automating chromatin-state discovery and characterization. *Nat Methods* 9, 215-216
2. Grant, C. E., Bailey, T. L., and Noble, W. S. (2011) FIMO: scanning for occurrences of a given motif. *Bioinformatics* **27**, 1017-1018
3. McLean, C. Y., Bristor, D., Hiller, M., Clarke, S. L., Schaar, B. T., Lowe, C. B., Wenger, A. M., and Bejerano, G. (2010) GREAT improves functional interpretation of cis-regulatory regions. *Nat Biotechnol* **28**, 495-501
4. Gilbert, N., and Labuda, D. (1999) CORE-SINEs: eukaryotic short interspersed retroposing elements with common sequence motifs. *Proc Natl Acad Sci U S A* **96**, 2869-2874
5. Rohrmoser, M., Kluge, M., Yahia, Y., Gruber-Eber, A., Maqbool, M. A., Forne, I., Krebs, S., Blum, H., Greifenberg, A. K., Geyer, M., Descostes, N., Imhof, A., Andrau, J. C., Friedel, C. C., and Eick, D. MIR sequences recruit zinc finger protein ZNF768 to expressed genes. *Nucleic Acids Res* **47**, 700-715
6. Chuong, E. B., Elde, N. C., and Feschotte, C. (2016) Regulatory evolution of innate immunity through co-option of endogenous retroviruses. *Science* **351**, 1083-1087
7. Franco, H. L., Nagari, A., and Kraus, W. L. (2015) TNFalpha signaling exposes latent estrogen receptor binding sites to alter the breast cancer cell transcriptome. *Mol Cell* **58**, 21-34
8. Theodorou, V., Stark, R., Menon, S., and Carroll, J. S. (2012) GATA3 acts upstream of FOXA1 in mediating ESR1 binding by shaping enhancer accessibility. *Genome Res* **23**, 12-22
9. Woodfield, G. W., Chen, Y., Bair, T. B., Domann, F. E., and Weigel, R. J. (2010) Identification of primary gene targets of TFAP2C in hormone responsive breast carcinoma cells. *Genes Chromosomes Cancer* **49**, 948-962
10. Malovannaya, A., Lanz, R. B., Jung, S. Y., Bulynko, Y., Le, N. T., Chan, D. W., Ding, C., Shi, Y., Yucer, N., Krenciute, G., Kim, B. J., Li, C., Chen, R., Li, W., Wang, Y., O'Malley, B. W., and Qin, J. (2011) Analysis of the human endogenous coregulator complexome. *Cell* **145**, 787-799
11. Xu, J., Liao, L., Ning, G., Yoshida-Komiya, H., Deng, C., and O'Malley, B. W. (2000) The steroid receptor coactivator SRC-3 (p/CIP/RAC3/AIB1/ACTR/TRAM-1) is required for normal growth, puberty, female reproductive function, and mammary gland development. *Proc Natl Acad Sci U S A* **97**, 6379-6384
12. Burwinkel, B., Wirtenberger, M., Klaes, R., Schmutzler, R. K., Grzybowska, E., Forsti, A., Frank, B., Bermejo, J. L., Bugert, P., Wappenschmidt, B., Butkiewicz, D., Pamula, J., Pekala, W., Zientek, H., Mielzynska, D., Siwinska, E., Bartram, C. R., and Hemminki, K. (2005) Association of NCOA3 polymorphisms with breast cancer risk. *Clin Cancer Res* **11**, 2169-2174
13. Morimoto-Tomita, M., Uchimura, K., Bistrup, A., Lum, D. H., Egeblad, M., Boudreau, N., Werb, Z., and Rosen, S. D. (2005) Sulf-2, a proangiogenic heparan sulfate endosulfatase, is upregulated in breast cancer. *Neoplasia* **7**, 1001-1010
14. Wang, X. (2014) An Exploration of Mutation Status of Cancer Genes in Breast Cancers. *Journal of Next Generation Sequencing & Applications* **1**, 103
15. Nassa, G., Tarallo, R., Guzzi, P. H., Ferraro, L., Cirillo, F., Ravo, M., Nola, E., Baumann, M., Nyman, T. A., Cannataro, M., Ambrosino, C., and Weisz, A. (2011) Comparative analysis of nuclear estrogen receptor alpha and beta interactomes in breast cancer cells. *Mol Biosyst* **7**, 667-676
16. Askarian-Amiri, M. E., Crawford, J., French, J. D., Smart, C. E., Smith, M. A., Clark, M. B., Ru, K., Mercer, T. R., Thompson, E. R., Lakhani, S. R., Vargas, A. C., Campbell, I. G., Brown, M. A., Dinger, M. E., and Mattick, J. S. (2011) SNORD-host RNA Zfas1 is a regulator of mammary development and a potential marker for breast cancer. *RNA* **17**, 878-891
17. Lee, K., Gjorevski, N., Boghaert, E., Radisky, D. C., and Nelson, C. M. (2011) Snail1, Snail2, and E47 promote mammary epithelial branching morphogenesis. *Embo J* **30**, 2662-2674

18. Grimm, S. L., and Rosen, J. M. (2003) The role of C/EBPbeta in mammary gland development and breast cancer. *J Mammary Gland Biol Neoplasia* **8**, 191-204
19. Robinson, G. W., Johnson, P. F., Hennighausen, L., and Sterneck, E. (1998) The C/EBPbeta transcription factor regulates epithelial cell proliferation and differentiation in the mammary gland. *Genes Dev* **12**, 1907-1916
20. Seagroves, T. N., Krnacik, S., Raught, B., Gay, J., Burgess-Beusse, B., Darlington, G. J., and Rosen, J. M. (1998) C/EBPbeta, but not C/EBPalpha, is essential for ductal morphogenesis, lobuloalveolar proliferation, and functional differentiation in the mouse mammary gland. *Genes Dev* **12**, 1917-1928
21. Milani, E. S., Brinkhaus, H., Dueggeli, R., Klebba, I., Mueller, U., Stadler, M., Kohler, H., Smalley, M. J., and Bentires-Alj, M. (2013) Protein tyrosine phosphatase 1B restrains mammary alveologenesis and secretory differentiation. *Development* **140**, 117-125
22. Julien, S. G., Dube, N., Read, M., Penney, J., Paquet, M., Han, Y., Kennedy, B. P., Muller, W. J., and Tremblay, M. L. (2007) Protein tyrosine phosphatase 1B deficiency or inhibition delays ErbB2-induced mammary tumorigenesis and protects from lung metastasis. *Nat Genet* **39**, 338-346
23. Barlund, M., Monni, O., Weaver, J. D., Kauraniemi, P., Sauter, G., Heiskanen, M., Kallioniemi, O. P., and Kallioniemi, A. (2002) Cloning of BCAS3 (17q23) and BCAS4 (20q13) genes that undergo amplification, overexpression, and fusion in breast cancer. *Genes Chromosomes Cancer* **35**, 311-317
24. Ruan, Y., Ooi, H. S., Choo, S. W., Chiu, K. P., Zhao, X. D., Srinivasan, K. G., Yao, F., Choo, C. Y., Liu, J., Ariyaratne, P., Bin, W. G., Kuznetsov, V. A., Shahab, A., Sung, W. K., Bourque, G., Palanisamy, N., and Wei, C. L. (2007) Fusion transcripts and transcribed retrotransposed loci discovered through comprehensive transcriptome analysis using Paired-End diTags (PETs). *Genome Res* **17**, 828-838
25. Gertz, J., Savic, D., Varley, K. E., Partridge, E. C., Safi, A., Jain, P., Cooper, G. M., Reddy, T. E., Crawford, G. E., and Myers, R. M. (2013) Distinct properties of cell-type-specific and shared transcription factor binding sites. *Mol Cell* **52**, 25-36
26. Brunelle, M., Nordell Markovits, A., Rodrigue, S., Lupien, M., Jacques, P. E., and Gevry, N. (2015) The histone variant H2A.Z is an important regulator of enhancer activity. *Nucleic Acids Res* **43**, 9742-9756
27. ENCODE_Project_Consortium (2012) An integrated encyclopedia of DNA elements in the human genome. *Nature* **489**, 57-74
28. Nautiyal, J., Steel, J. H., Mane, M. R., Oduwole, O., Poliandri, A., Alexi, X., Wood, N., Poutanen, M., Zwart, W., Stingl, J., and Parker, M. G. (2013) The transcriptional co-factor RIP140 regulates mammary gland development by promoting the generation of key mitogenic signals. *Development* **140**, 1079-1089
